# A combined fragment-based virtual screening and STD-NMR approach for the identification of E-cadherin ligands

**DOI:** 10.3389/fchem.2022.946087

**Published:** 2022-08-19

**Authors:** Francesca Vasile, Francesca Lavore, Silvia Gazzola, Chiara Vettraino, Emilio Parisini, Umberto Piarulli, Laura Belvisi, Monica Civera

**Affiliations:** ^1^ Department of Chemistry, Università Degli Studi di Milano, Milan, Italy; ^2^ Department of Science and High Technology, Università Degli Studi Dell’Insubria, Como, Italy; ^3^ Center for Nano Science and Technology, Istituto Italiano di Tecnologia @Polimi, Milan, Italy; ^4^ Latvian Institute of Organic Synthesis, Riga, Latvia; ^5^ Department of Chemistry “Giacomo Ciamician”, Università Degli Studi di Bologna, Bologna, Italy

**Keywords:** fragment virtual screening, molecular dynamics, STD-NMR, cadherins, protein–protein interaction (PPI)

## Abstract

Cadherins promote cell-cell adhesion by forming homophilic interactions *via* their N-terminal extracellular domains. Hence, they have broad-ranging physiological effects on tissue organization and homeostasis. When dysregulated, cadherins contribute to different aspects of cancer progression and metastasis; therefore, targeting the cadherin adhesive interface with small-molecule antagonists is expected to have potential therapeutic and diagnostic value. Here, we used molecular docking simulations to evaluate the propensity of three different libraries of commercially available drug-like fragments (nearly 18,000 compounds) to accommodate into the Trp2 binding pocket of E-cadherin, a crucial site for the orchestration of the protein’s dimerization mechanism. Top-ranked fragments featuring five different aromatic chemotypes were expanded by means of a similarity search on the PubChem database (Tanimoto index >90%). Of this set, seven fragments containing an aromatic scaffold linked to an aliphatic chain bearing at least one amine group were finally selected for further analysis. Ligand-based NMR data (Saturation Transfer Difference, STD) and molecular dynamics simulations suggest that these fragments can bind E-cadherin mostly through their aromatic moiety, while their aliphatic portions may also diversely engage with the mobile regions of the binding site. A tetrahydro-β-carboline scaffold functionalized with an ethylamine emerged as the most promising fragment.

## Introduction

Protein–protein interactions (PPIs) are crucial events that play a significant role in many physiological and pathological processes. While in recent years PPIs have received increasing attention as attractive pharmaceutical targets, their actual druggability is often hindered by their large and usually featureless binding interface, which is typically formed by non-continuous hot spots. Indeed, only a limited number of successful PPI drug discovery campaigns have been reported to date (Lu et al., 2020).

Among the various approaches that are used for the discovery of PPI modulators, fragment-based drug discovery (FBDD) has proved particularly effective for the identification of small-molecule hits (Li, 2020). Fragments have a low molecular weight and can bind regions that are often hard to target, such as allosteric sites or hot-spot residues. Initial fragment hits generally show a weak binding affinity, usually in the µM–mM range since they possess few atoms that can form stabilizing interactions with the surface of the target protein (de Souza Neto et al., 2020). However, compared to traditional libraries, fragment-based libraries have the advantage of covering a broader chemical space and consist of a relatively small number of molecules. Additionally, fragment libraries typically comprise organic compounds that possess good pharmacokinetic properties and can provide great opportunities for further chemical derivatization. Hence, they usually represent an optimal starting point for the development of larger drug-like molecules (de Souza Neto et al., 2020).

**FIGURE 1:**
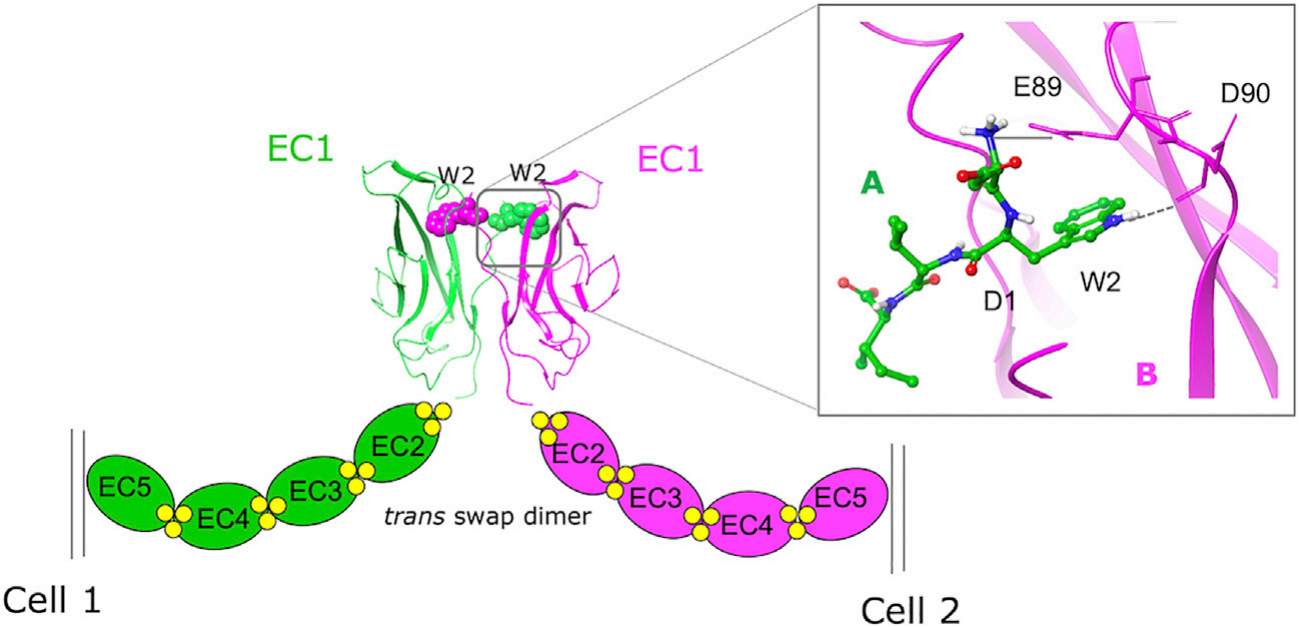
A schematic representation of the homophylic interaction between type I cadherins extracellular domains. The W2 residues of the EC1 domains protruding from opposing cells are mutually exchanged (green = monomer A, purple = monomer B) to form the swapped dimer. An expanded view of the swap dimer interface of E-cadherin (2O72.pdb) with the key interactions formed by the N-terminal sequence of monomer A in the hydrophobic pocket of monomer B is also shown.

Both *in silico* and experimental FBDD approaches have been taken to identify potential modulators of PPIs involving cadherins (Senoo et al., 2021). Cadherins are transmembrane cell adhesion proteins that mediate adherens junction formation *via* a dynamic homodimerization mechanism whereby the N-terminal extracellular portions of cadherins protruding from opposing cells interact with each other and take part in a complex conformational equilibrium characterized by multiple transient states featuring different binding interfaces (Shapiro et al., 1995; Chen et al., 2005; Brasch et al., 2012; Doro et al., 2015b). In classical cadherins, which are a subset of the large cadherin superfamily, the entire ectodomain comprises five tandemly arranged extracellular cadherin (EC) repeats. Its overall conformation is rigidified and maintained functionally active by the presence of three calcium ions at each of the interdomain junctions. By interacting with α- and β-catenin, the cadherin cytoplasmic tail connects the protein on the surface of the cell to the actin cytoskeleton. In classical cadherins, the endpoint of the homodimerization mechanism consists in the mutual exchange of the 6-residue long N-terminal sequence of the protein, the so-called “adhesion arm.” As revealed by X-ray structures (Parisini et al., 2007; Harrison et al., 2011), in type I cadherins the Trp2 side chain of the adhesion arm inserts into the hydrophobic pocket of a partner molecule, leading to the formation of a strand-swap homodimer (Figure 1). Overall, cadherins cluster at the adherens junctions to form dynamic zipper-like structures whose plasticity is crucial for conferring cadherins their adhesive functions and mechanotransduction properties.

E-cadherin, the prototypical type I classical cadherin, is mainly expressed in epithelial tissues. Primarily considered a tumor suppressor protein, E-cadherin is often downregulated during the epithelial to mesenchymal transition (EMT) process that occurs in cancer cells (Yang and Weinberg, 2008; Mendonsa et al., 2018), which leads to the detachment of the cancerous cell from the primary tumor site. Studies have shown that E-cadherin expression is maintained in carcinomas and distal metastases, as observed for instance in late-stage tumors in epithelial ovarian cancer (EOC) (Roggiani et al., 2016).

In this context, the discovery of ligands targeting cadherin homophilic interactions could help elucidate their role during tumor growth, invasion, and metastasis (Kaszak et al., 2020). However, the dynamic nature of the homophilic interface, the existence of multiple conformational intermediates, and the complexity of the cadherin dimerization mechanism make the design of small cadherin ligands challenging.

We have recently reported a library of peptidomimetic ligands that mimic the natural sequence DWVI, a sizeable portion of the whole E- and the N-cadherin adhesion arm (Doro et al., 2015a). These ligands were designed to target the Trp2 binding site by replacing the central dipeptide unit WV with different chemical scaffolds bearing an aromatic moiety. A ligand from that library, FR159, was found to be able to inhibit E-cadherin-mediated cell-cell adhesion at low millimolar concentrations and better than ADH-1 (Exherin), a small cyclic peptide that had previously entered clinical trials in cancer patients (Perotti et al., 2009). While these compounds were designed to bind into the Trp2 pocket, the crystal structure of the complex between a truncated form of E-cadherin-EC1-EC2 (lacking Val1 and Trp2) and FR159 (PDB code 4ZTE) showed that the compound binds into a hydrophobic pocket that is transiently formed when the protein assumes the X-dimer conformation, a crucial intermediate that promotes strand-swap dimer formation (Nardone et al., 2016). Based on this crystal structure, a virtual screening approach with commercially available compounds led to the subsequent identification of two inhibitors of E-cadherin-mediated cell-cell adhesion that are active at 50 µM (Dalle Vedove et al., 2019). Interestingly, STD-NMR experiments have shown that the binding epitope of FR159 changes depending on whether intact E-cadherin-EC1-EC2 or the truncated form of the protein lacking the first two N-terminal residues is used (Civera et al., 2019), also showing a temperature modulation. Molecular dynamics simulations have suggested the possibility that, in addition to the binding conformation observed in the crystal structure, FR159 may also bind to the Trp2 pocket explaining these NMR data. In this scenario, the highly flexible adhesion arm could be engaged in the interaction with FR159, suggesting that FR159 may bind to E-cadherin in different possible conformations.

In this work, we took a fragment-based virtual screening approach in combination with molecular dynamics simulations and STD-NMR measurements to target the Trp2 binding pocket of E-cadherin and identify ligands that can fit into this site. The results might improve our understanding of the cadherin homophilic interaction mechanism and contribute to the design of novel diagnostic or therapeutic agents. In particular, by docking calculations, we screened three libraries of commercially available drug-like fragments and performed a similarity search on PubChem database starting from the docking best-ranked fragments. We then selected seven fragments displaying five different chemotypes for further molecular dynamics simulations and experimental validation *via* STD-NMR measurements.

All the identified fragments bind to the intact E-cadherin-EC1-EC2 construct *via* their aromatic moieties. Molecular dynamics simulations suggest a pivotal role played by the adhesion arm, which could help stabilize the fragments inside the Trp2 pocket and contribute to the elucidation of the signals observed in the STD spectra.

## Results and discussion

### Fragment-based virtual screening in the Trp2 pocket

**FIGURE 2:**
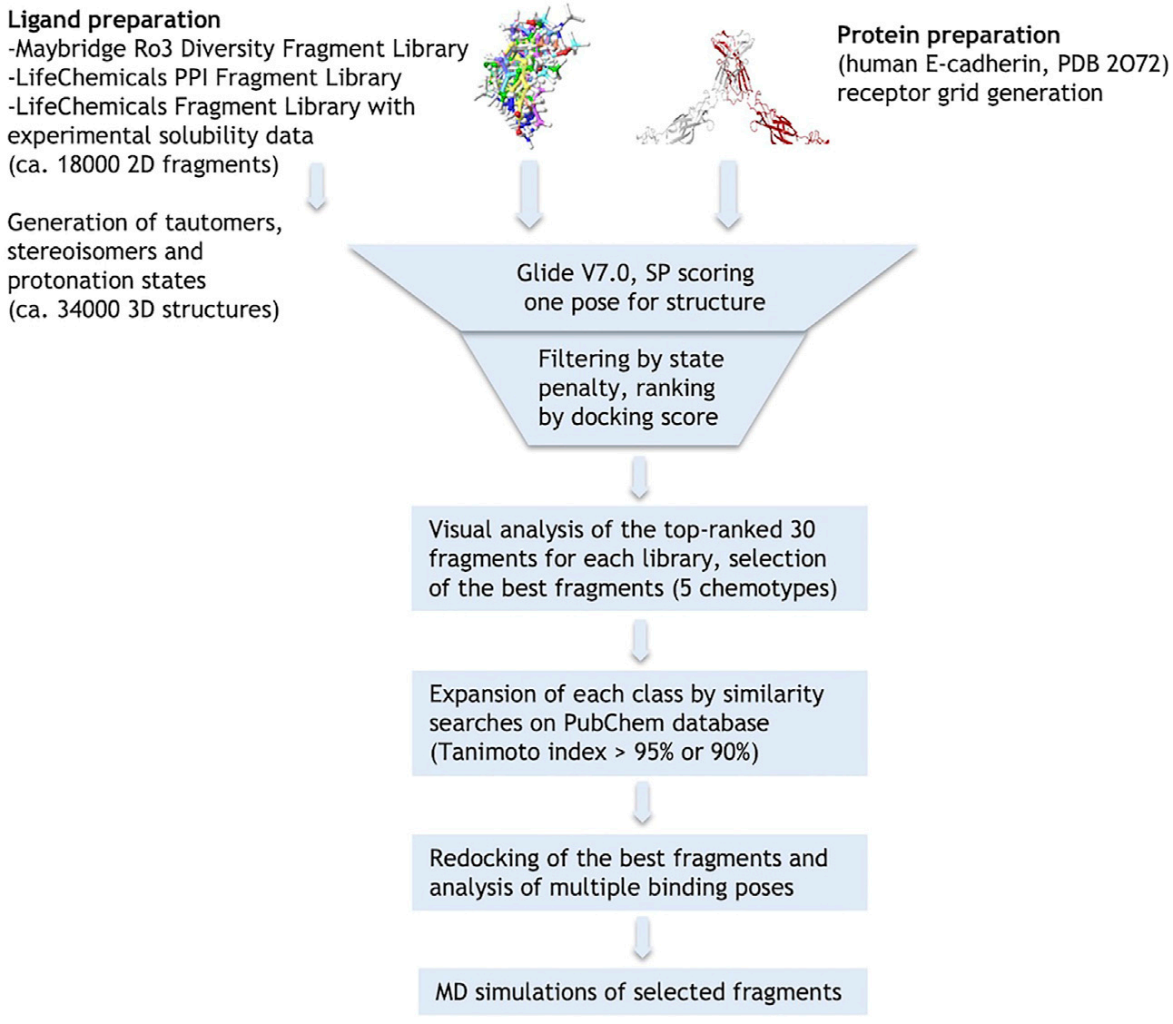
Schematic representation of the virtual screening workflow.

**FIGURE 3:**
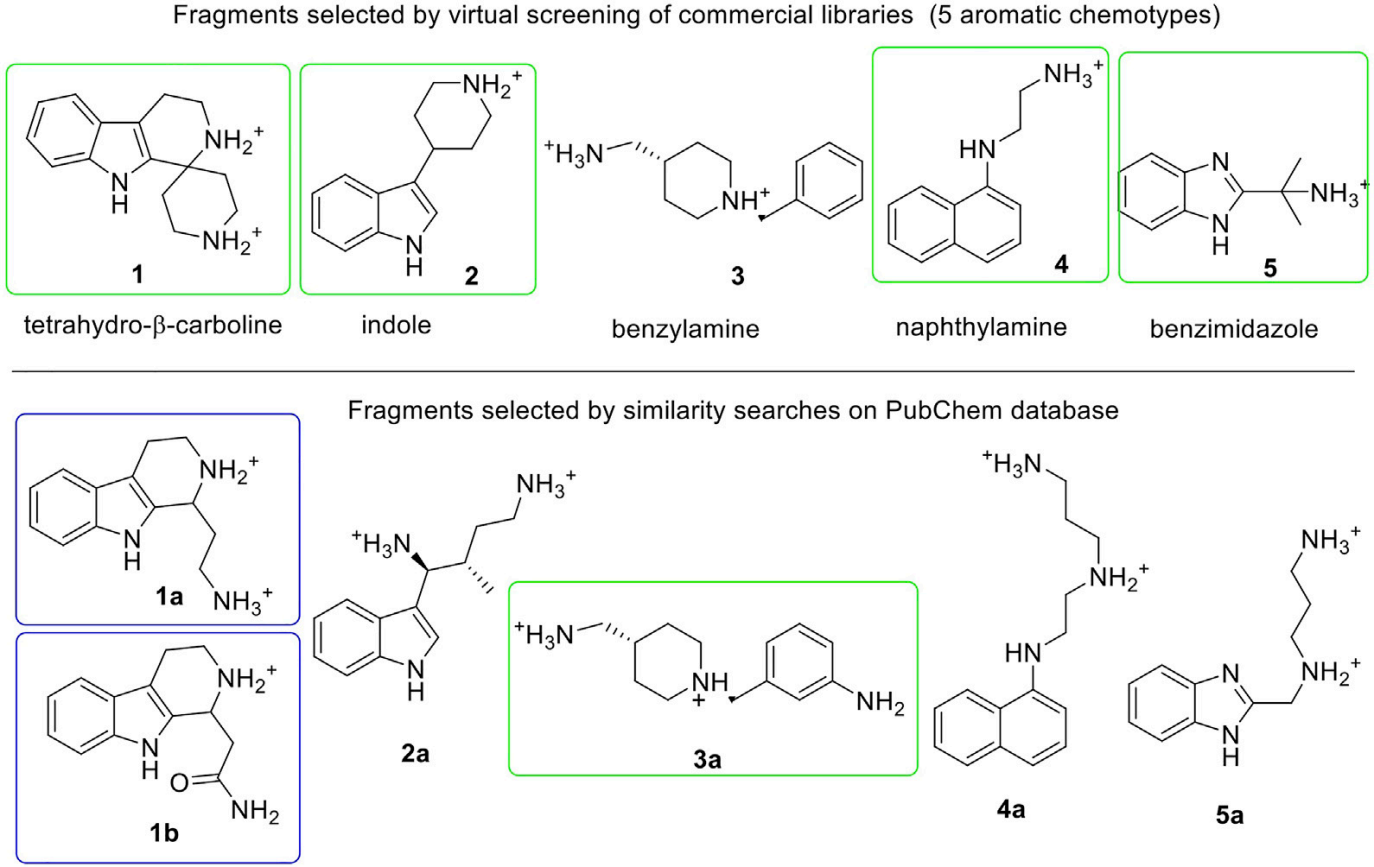
Fragments selected by virtual screening of commercial libraries (top section) and by similarity searches on PubChem database (bottom section). Synthetized or purchased compounds for NMR experiments are shown in blue and green boxes, respectively. Only the enantiomer *S*-**1a** was found by similarity search. Fragments are displayed in their most favorable protonation state.

The crystallographic structure of the human E-cadherin strand-swap dimer (PDB code 2O72) (Parisini et al., 2007) was prepared as described in the *Material and Methods* section and used to define a docking model centered on the Trp2 pocket of the EC1 domain. Three libraries of commercially available drug-like fragments (“Maybridge Ro3 Diversity Fragment Library,” “LifeChemicals PPI Fragment Library” and “LifeChemicals Fragment Library with Experimental Solubility Data,” for a total of nearly 18,000 2D fragments) were retrieved for *in silico* screening according to the workflow shown in Figure 2, and processed with the Schrödinger Ligprep utility to generate tautomers, stereoisomers and protonation states at pH 7 ± 2. The calculation provided approximately 34,000 3D structures that were docked into the E-cadherin model (Glide SP, v7.0) (Friesner et al., 2004) saving one pose for each run. Docking poses were filtered on the basis of their state penalty to exclude unfavorable tautomeric and protonation states and then ranked according to the Glide score. The binding mode of the top 30 fragments of each library was carefully analyzed to identify the fragments that are able to fit into the Trp2 pocket and to form additional interactions within the pocket. Five different aromatic chemotypes emerged from this analysis: except for the simple benzene ring, all the classes proved capable of reproducing the interactions of the Trp2 side chain in the strand-swap dimer pocket, including the H-bond with the backbone carbonyl group of Asp90. The structures of the top-ranked fragments featuring the five different chemotypes are shown in Figure 3 (top section). In addition to the aromatic scaffold, the fragments are equipped with an aliphatic handle bearing at least one amine moiety, which can engage in salt bridge interactions with the protein, likely mimicking the N-terminal amino group of the adhesion arm.

### Hit-fragment expansion

The chemical space around each fragment chemotype was further explored by performing similarity searches in the PubChem database (Tanimoto index >95% or 90%) starting from the best fragments identified in the previous step. The 2D structures of commercially available compounds resulting from each search (overall about 3,200 2D structures) were converted into 3D structures (for a total of nearly 9,600 structures) and then docked in the E-cadherin model following the protocol described above. After filtering by state penalty, the binding modes of the top 20 fragments of each run were analyzed to identify optimized fragments able to improve their interactions with the Trp2 pocket and the neighboring residues involved in the strand-swap dimer, while maintaining low stereochemical complexity (Figure 3, bottom section). Other factors like commercial availability, purchasing cost and synthetic feasibility were taken into consideration when we selected fragments for experimental validation.

### Re-docking of the selected fragments

**FIGURE 4:**
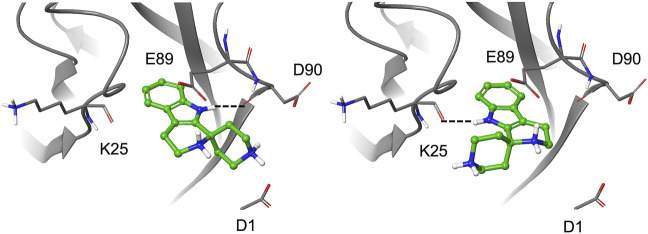
Docking pose A (left) and pose B (right) for fragment **1**.

To further explore the binding of the fragments in the hydrophobic pocket and assess poses stability, the selected fragments were re-docked into the E-cadherin model saving 10 poses per fragment and using an improved version of the OPLS force field (OPLS3, Harder et al., 2016). For most fragments, the analysis of the docking poses highlighted two main binding modes, named type A and type B, differing in the orientation of the aromatic moiety within the Trp2 pocket. In particular, the type A binding mode is characterized by the H-bond between the heterocyclic or aniline NH moiety and the backbone carbonyl group of Asp90, mimicking the most commonly observed native H-bond of the Trp2 indole in the strand-swap dimer. In the type B binding mode, the aromatic moiety is kept in the pocket but it is oriented to form an H-bond with the backbone carbonyl group of Lys25, as observed in the crystal structure of human P-cadherin-EC1-EC2 (Dalle Vedove et al., 2015) (Figure 4).

It is worth noting that for the *S*-enantiomer of fragment **1a** and for fragment **4**, only type A poses are obtained, while a preference for type A poses with a minor contribution from the type B binding mode is observed for the *R*-enantiomer of **1a** and **1b** and for fragments **1** and **5**. A type B best pose is found for the *S*-enantiomer of **1b** and for fragments **2** and **3a,** whose saved poses alternate between type A and B. According to the Glide score values of the top-ranked poses, *S*-**1a** is the best ligand (−9.73 kcal/mol) with a ligand efficiency of −0.608 kcal/mol [calculated as (docking score)/(number of heavy atoms)].

In most poses, the amine moieties of all the fragments are engaged in salt bridges with the negatively charged side chains of residues in the pocket (Glu89, Asp90) and/or in the adhesion arm (Asp1). Details on docking results and poses selected for MD simulations are provided in the Supplementary Material section. All the fragments selected for experimental validation were purchased from commercial vendors except fragments **1a** and **1b,** which we synthesized as racemic mixtures (see Supplementary Material).

### STD-NMR experiments

Using STD-NMR spectroscopy, we studied the interaction between the fragments and the two-domains EC1-EC2 construct of human E-cadherin, the minimal functional subunit that binds calcium and forms homodimers (Davila et al., 2018). NMR experiments were performed in 20 mM deuterated phosphate buffer pH 7.4, 150 mM NaCl and 1 mM CaCl_2_ and at a protein concentration of 20 μM. At this protein concentration, E-cadherin is predominantly monomeric, while dimeric forms or even oligomers are present in solution at concentrations higher than 40 µM (Davila et al., 2018).

STD is one of the main NMR techniques that is used for studying ligand-protein interactions (Mayer and Meyer, 1999; Mayer and Meyer 2001). It is based on the observation of the NMR signals of the ligand and it allows the identification of its binding epitope (Meyer and Peters, 2003; Gatti et al., 2014; Sattin et al., 2016).

To obtain an STD-NMR spectrum, the protein is selectively irradiated in a region in which only the frequencies of the protein and not those of the ligand are present. As a result, at the ligand-binding site, magnetization is transferred from the protein to the ligand, which can then be detected. Indeed, the NMR signals from the protein will not be observed, as the technique operates under a large excess of the ligand. By integrating the STD spectrum, it is possible to define the absolute STD percentage, a value that reflects the proximity of the protons to the protein (short protein–ligand distances produce a strong intensity of the corresponding STD signal), thus allowing the mapping of the ligand’s epitope. Additionally, relative STD percentages can be calculated by normalizing all measured STD intensities against the most intense signal, which is arbitrarily assigned a value of 100%. The group epitope mapping thus obtained reveals which chemical moieties of the ligand are key for molecular recognition in the binding site.

**FIGURE 5:**
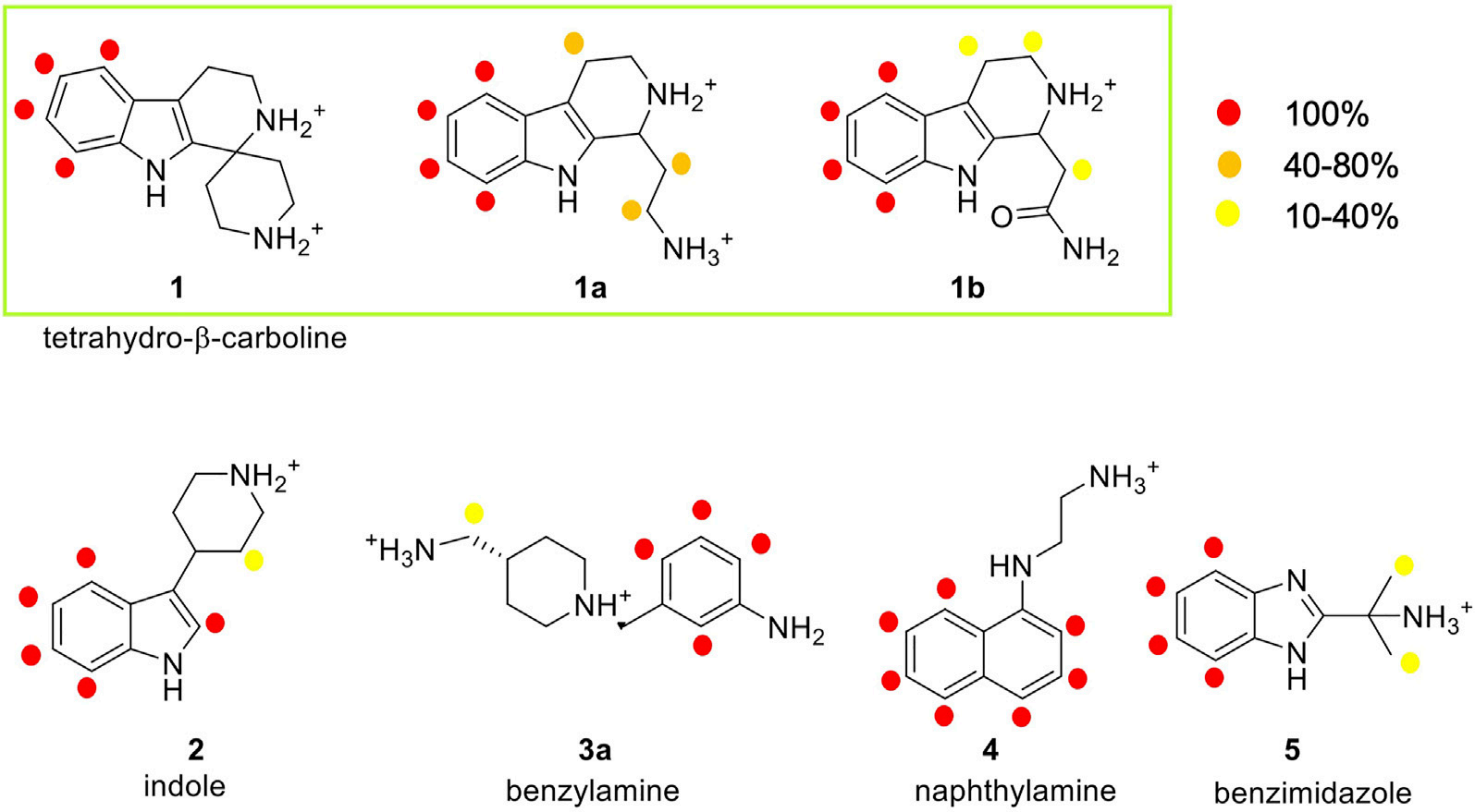
Analysis of the STD experiments for the seven selected fragments with the E-cadherin-EC1-EC2 construct. STD spectra were acquired irradiating at −0.1 ppm and with a saturation time of 2.94 s. For each fragment, the relative STD effects are conveyed by color code: 100% relative STD is reported in red, values between 40 and 80% in orange, and values between 1 and 40% in yellow.

In this work, STD experiments were done in order to assess the possible interaction of the seven selected fragments with human E-cadherin-EC1-EC2. The ^1^H and STD spectra of the fragments in the presence of the protein are reported in the Supplementary Figures S5–S11. Despite the small size of the fragments, epitope mapping was possible for all the hit fragments, allowing the identification of their protons that are involved in protein binding. The NMR results are summarized in Figure 5, where the relative STD intensities are reported using a color code.

In the spectrum of fragment **1**, which contains a tetrahydro-β-carboline linked to a piperidine ring through a spiro carbon, only the aromatic protons of the carboline moiety appear to interact with the protein, while no NMR signals relate to the piperidine moiety.

Fragment **1a**, analyzed as a racemate, contains the tetrahydro-β-carboline ring (as in fragment **1**) linked to an ethylamine group. Its STD spectrum allows the determination of the epitope that involves all the moieties of the molecule. Indeed, its binding appears to be driven by both the aromatic and the aliphatic protons of the tetrahydro-β-carboline and by the ethylamine chain. The relative STD percentages are high also in the case of the CH_2_ groups, suggesting a good and stable interaction of the entire fragment.

Fragment **1b**, also analyzed as a racemate in the experiments, differs from **1a** for the presence of an amide group instead of the primary amine. This change affects the binding properties of the molecule and, in fact, the STD spectrum reveals strong signals for the aromatic group and very low-intensity peaks for the aliphatic protons. This suggests a strong binding of the aromatic ring in the interaction and a lower involvement of the amide side chain.

Fragment **2** contains an indole ring connected to piperidine and, as evidenced in its STD spectrum in the presence of E-cadherin, both groups are involved in the interaction.

Considering the spectrum of fragment **3a,** which contains an aniline linked to a piperidine, interaction with the protein can be observed for all the aromatic protons of the aniline and for the methylene group connecting the primary amine to the piperidine ring (Supplementary Figure S9).

In the case of fragment **4,** only the aromatic protons of the naphthalene moiety are part of the binding epitope, while for fragment **5** both aromatic protons of the benzimidazole core and aliphatic protons of the methyl substituents are found in the epitope.

Our STD results clearly indicate that all the hit fragments can bind E-cadherin and that the binding is mostly driven by the aromatic moieties of the ligands. Although it is known that a source of minor differences in epitope maps can be the presence of D_2_O, as the polar group has their exchangeable protons replaced by ^2^H, which is inefficient for transferring saturation (Monaco et al., 2017), a modulation is also observed for the aliphatic moieties, and in particular:

- no interaction is detected for the piperidine ring and for ethylamine in fragments **1** and **4**, respectively
- very weak interactions are observed for the piperidine ring of fragment **2**, for the CH_3_ groups of fragment **5,** and also for the CH_2_ groups of fragments **1b** and **3a**
- a strong interaction is detected for the CH_2_ groups of **1a**, indicating a relatively strong and stable binding with the protein and suggesting that this may be the most promising fragment of our series.

### Molecular dynamics simulations

Starting from the most representative docking poses of the seven fragments, MD simulations were carried out to allow protein flexibility [Desmond, 500 ns, NVT, T = 300 K, OPLS3e force field (Roos et al., 2019), dt = 2 fs, SPC water model (Berweger et al., 1995)] and help the interpretation of the NMR results. For each run, 5,000 structures were sampled for the analysis.

The analysis of the binding modes and fragment interactions fit well with the STD-NMR data reported above. Here, we will focus our discussion on the tetrahydro-β-carboline fragments **1**, **1a,** and **1b**, whereas fragments **2, 3a, 4,** and **5** are reported and discussed in the Supplementary Material section. In particular, we will discuss the interactions formed by the substituent during the simulations and compare the results with the interactions observed in the STD spectra considering that the replacement of the terminal amine (as in **1a**) with a neutral amide (**1b**) or its rigidification into a cyclic amine (**1**) modulates the corresponding STD signals.

Fragment **1a** is the only compound of the tetrahydro-β-carboline series that shows STD signals of comparable intensity for both the aliphatic and the aromatic protons.

In the NMR experiment, the compound is a racemic mixture and the detected signals could derive from one or both enantiomers. According to the simulation results, enantiomer *S*-**1a** seems to form more interactions with the protein, especially for the terminal amine, which has the proper orientation to engage both the Asp1 residue of the adhesion arm and the residues in the pocket in stable salt bridges.

**FIGURE 6:**
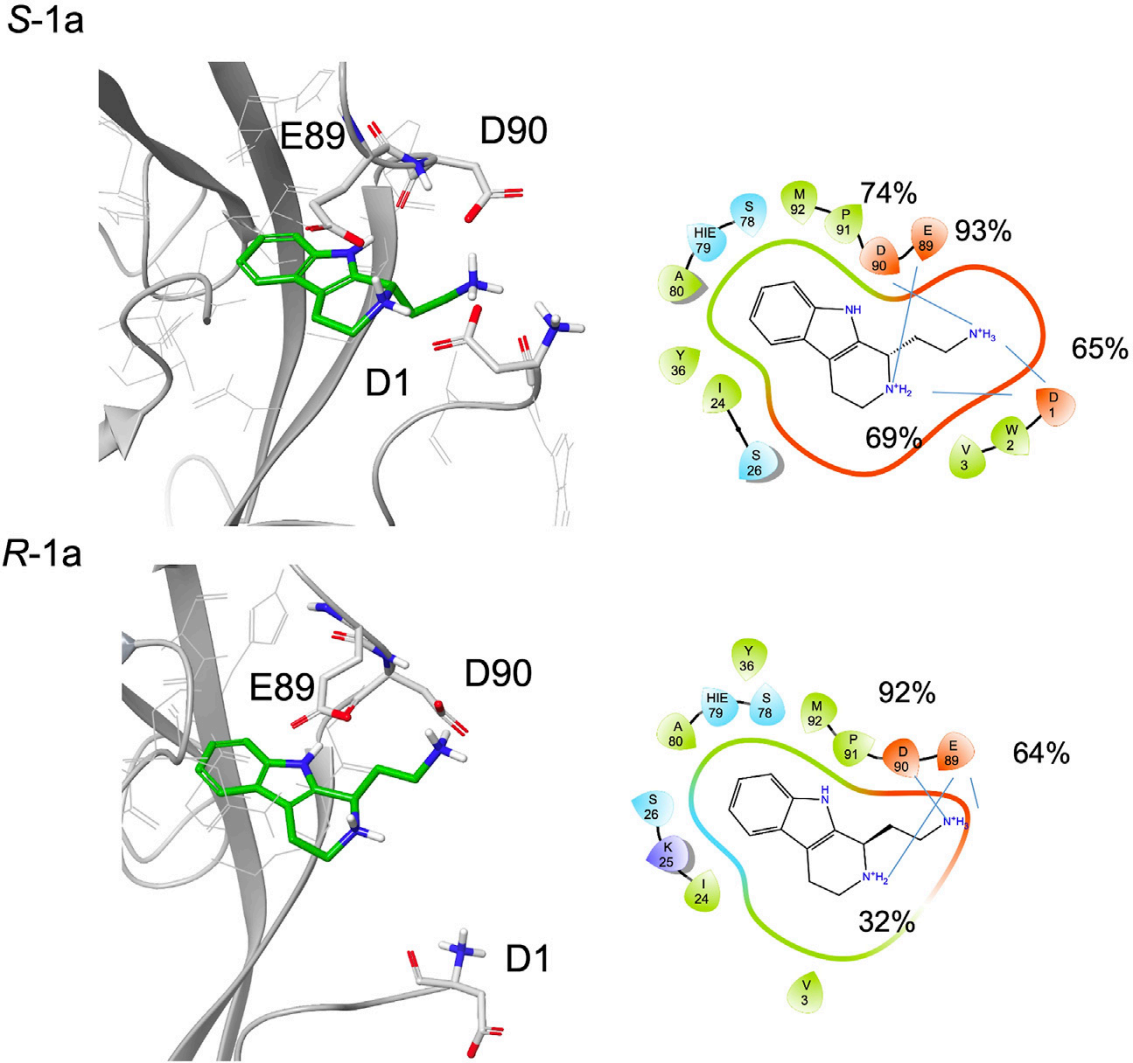
A snapshot from MD simulation for *S*-**1a** and *R*-**1a**. The corresponding 2D ligand interaction diagram with the percentages of salt bridges monitored during the MD simulations is also shown.

Indeed, starting from a type A pose, during the simulations both enantiomers maintain their ionic interactions with the residues Glu89 and Asp90 inside the pocket, while only *S*-**1a** forms additional salt bridges with the side chain of Asp1 (Figure 6, Supplementary Table S1). Furthermore, the aromatic moiety of *S*-**1a** is more firmly bound to the hydrophobic pocket than *R*-**1a**, which leaves the cavity in the last 40 ns (the hydrogen bond with the Asp90 backbone is 98% populated for *S*-**1a** and 88% for *R*-**1a**). Before exiting the cavity, *R*-**1a** forms interactions with residues Glu89 and Asp90, while no contacts with the residues of the adhesion arm are observed.

Compared to **1a**, **1b** is functionalized with an amide group on the cyclic amine and the STD spectrum of the racemic mixture shows lower intensities for the aliphatic protons than for the aromatic ones. The aromatic ring maintains its hydrogen bond with the backbone of Asp90 (97% for *S*-**1b**, 81% for *R*-**1b**) or Lys25 (70%, for *S*-**1b**).

**FIGURE 7:**
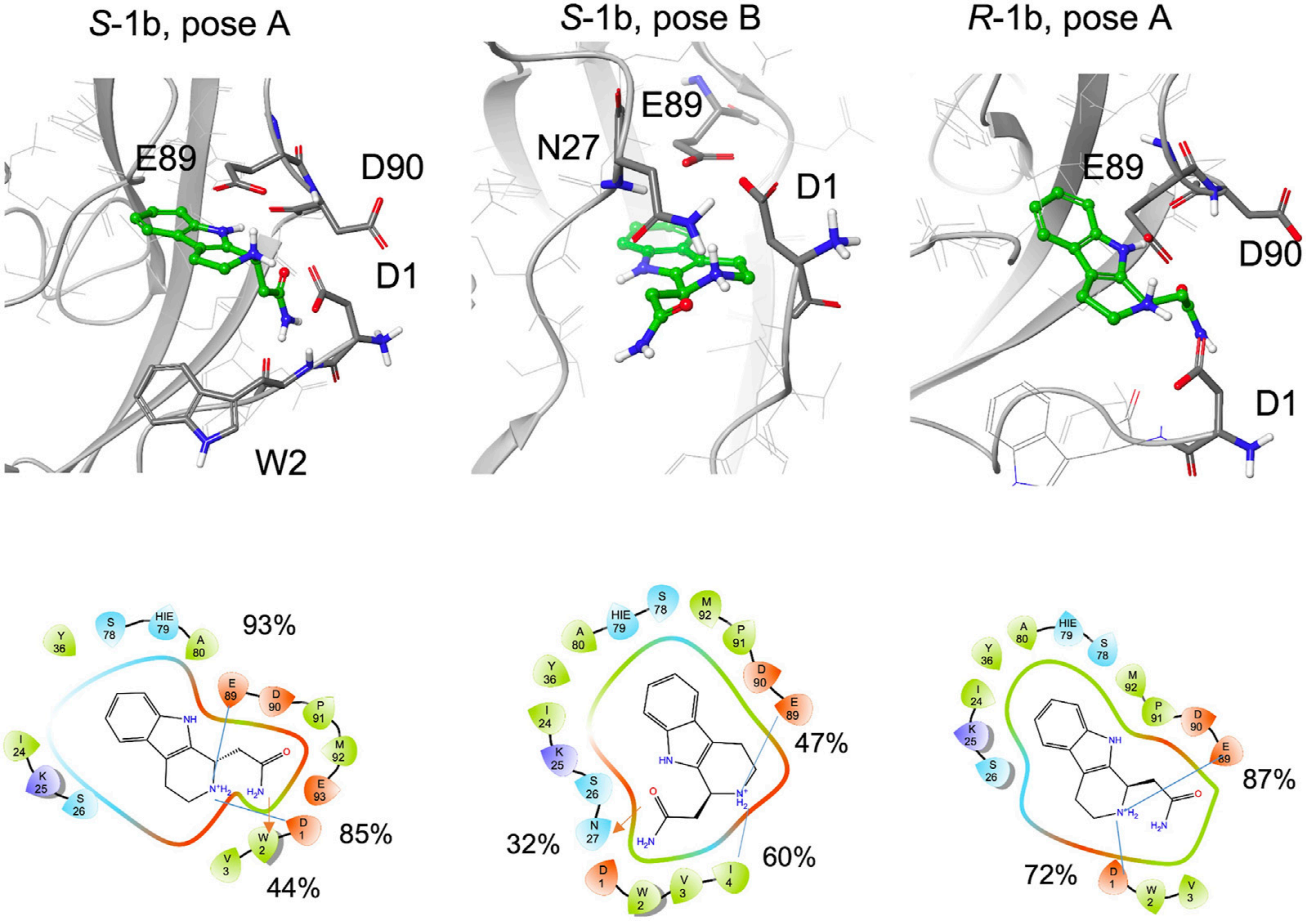
Snapshots from MD simulations:. Left *S*-**1b** pose A, middle *S*-**1b** pose B, right *R*-**1b** pose A. The corresponding 2D ligand interaction diagram with the percentages of salt bridges (blue line) and hydrogen bonds (red arrow) monitored during the MD simulations is also shown.

During the simulations, both enantiomers *S*-**1b** and *R*-**1b** maintain charge-charge interactions between the amine group and the side chains of Asp1 and Glu89 (Figure 7, Supplementary Table S2) while the different stereochemistry or binding mode affects the orientation of the amide group. For *R*-**1b** the amide group appears to be solvent exposed and forms no interactions with the protein, whereas for *S*-**1b** a hydrogen bond with the backbone of Trp2 is formed (44% populated). If we consider the simulation of *S*-**1b** starting from pose B**,** the amide moiety is oriented towards the Asn27 side chain and forms a hydrogen bond with it (32% populated). This binding mode leads to an unstable interaction of the aromatic ring which exits the hydrophobic pocket between 460 and 480 ns and re-enters in the last part of the simulation.

In general, compared to the charged ethylamine of **1a**, the neutral amide of **1b** seems to establish less populated interactions with the protein, in agreement with the differences observed in the STD-NMR spectra for the aliphatic signals.

**FIGURE 8:**
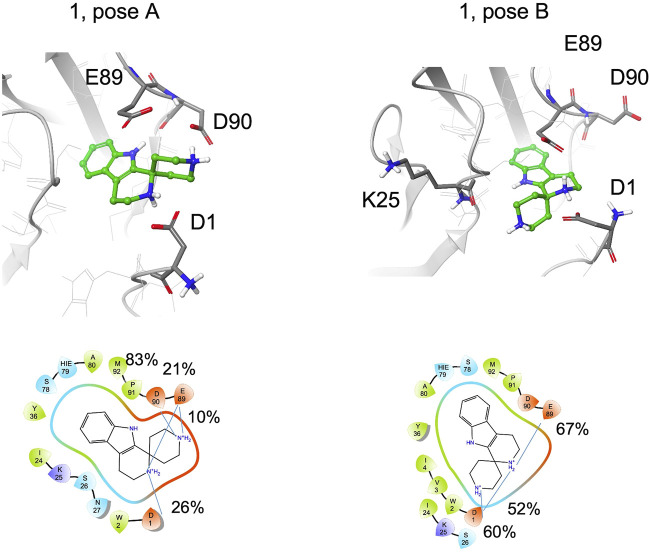
Snapshots from MD simulations for **1** (pose A and B). The corresponding 2D ligand interaction diagram and percentages of salt bridges monitored during the MD simulations are also shown.

Fragment **1** includes the terminal amine into a spiro cycle. Compared to **1a,** this rigidification leads to a loss of STD-NMR signals for the aliphatic protons. The aromatic ring remains bound to the site; for the pose A, hydrogen bond with the backbone of Asp90 is always formed, for pose B 77% of the structures form a hydrogen bond with the backbone of Lys25 and both amine groups form salt bridges with the side chain of Glu89, Asp90 or Asp1 (Figure 8, Supplementary Table S3). However, the possibility to engage both protein regions simultaneously and efficiently, as observed for *S*-**1a**, is lost. In particular, depending on the binding mode, the terminal amine prefers to interact with the residues in the pocket (Glu89, Asp90) or with Asp1 in the adhesion arm. By comparing the type A binding mode of **1** to that of *S*-**1a**, a drop in the percentage of contacts with the adhesive arm was observed (the salt bridge with Asp1 is 26% populated with respect to the two salt bridges of *S*-**1a,** which are 65% and 69% populated) and the cyclic amine is less engaged in the interaction with E-cadherin (for **1** the salt bridges percentage are of 26% with Asp1 and 21% with Glu89, for *S*-**1a** 69% and 93%, respectively, Supplementary Tables S1, S3). For the terminal amine, no interactions with the adhesive arm are formed for **1** while for *S*-**1a** contribution of Asp1 in stabilizing the ligand-binding mode was observed (65% of salt bridge formation).

The absence of aliphatic signals (which can be slightly underestimated, as discussed in the NMR STD-NMR experiments section) in STD spectra of **1** can be related to the low percentage of salt bridge formation observed in MD simulation with Asp1.

## Conclusion

Targeting cadherin–cadherin interactions with ligands that are potentially able to inhibit or modulate their adhesive function during tumor growth is a challenging goal. In this paper, we identified five different ligand chemotypes by performing a fragment-based virtual screening inside the Trp2 pocket of E-cadherin. Then, by means of molecular dynamics simulations and STD-NMR experiments, we analyzed seven fragments containing different aromatic scaffolds decorated with aliphatic amines. We assessed the binding propensity of all fragments and we identified the tetrahydro-β-carboline scaffold as the most promising. The analysis of the amine moiety suggests that a flexible substituent such as ethylamine allows for a better fit into the binding pocket and for the formation of stable salt bridges with the protein. On the other hand, the rigidification of the amine, which results from the introduction of a piperidine, and the substitution with a less basic substituent, such as an amide, reduce the interaction of the aliphatic portion of the ligand. Finally, it is possible that the adhesion arm, which is dynamically interacting with the protein, may also participate in the stabilization of the ligand inside the pocket. In this respect, it is interesting to observe that in our simulations all the fragments that show an STD signal of the aliphatic portion also engage this moiety in a salt bridge with the N-terminal group of Asp1.

It is known that fragments often represent small and essential cores for target binding and the identification of these favorable moieties is an important step for the design of larger molecules with higher binding affinity. These data will be used for further rounds of optimization, and we expect that the evolution of these fragments will afford compounds with higher affinity and activity towards E-cadherin.

## Materials and methods

### Computational studies

All the calculations were performed using the Schrödinger Suite through Maestro (version 2018-1) graphical interface.

### Ligand preparation

The Maybridge and LifeChemicals libraries of small fragments listed below were used for *in silico* screening. The LigPrep v3.7 tool was run to generate protonation states at pH 7 ± 2, tautomers, and stereoisomers. The calculations provided the following results: Maybridge Ro3 Diversity Fragment Library from 2,500 2D fragments to 4,290 3D structures, LifeChemicals PPI Fragment Library from 2,509 2D fragments to 6,187 3D structures, LifeChemicals Fragment Library with Experimental Solubility Data from 12,845 2D fragments to 23,540 3D structures.

### Model generation

The atomic coordinates of the crystal structure of the human E-cadherin-EC1-EC2 strand-swap dimer (PDB code 2O72) (Parisini et al., 2007) were retrieved from the Protein Data Bank. The EC1 domain (residues 1-103) of monomer A and the DWVI tetrapeptide sequence (residues 1-4) of monomer B were used to define the receptor-ligand complex. Two Ca^2+^ ions at the EC1-EC2 interface were maintained, while crystallographic water molecules were removed during the input preparation. The system was then prepared using the Protein Preparation Wizard of the Maestro graphical user interface (pKa was calculated for protein residues using the PROPKA method at pH 7.0) by optimizing the orientation of hydrogen bonds and charge interactions, followed by a restrained minimization of the whole system (0.30 Å of RMSD on heavy atoms) using the OPLS_2005 force field (Banks et al., 2005). The final refined structure was used to generate the docking receptor grid.

### Docking calculations

Automated docking calculations were performed using Glide (Grid-based Ligand Docking with Energetics) V7.014 (Friesner et al., 2004). The grid was generated for the E-cadherin structure prepared as described in the previous section and selecting the EC1 domain (1-103 residues) of monomer A as receptor and the DWVI tetrapeptide of monomer B as ligand. The center of the grid box was defined by the centroid of the DWVI sequence, while the size of the cubic inner box for placing the ligand center was set to 14 Å and the size of the cubic outer box to 26 Å. Docking calculations were performed using the standard precision mode (SP) and the OPLS_2005 force field. The receptor was considered as a rigid body while the flexible ligand docking approach was carried out with the option “Penalize nonplanar conformation” for amides. No Epik state penalties were used in the docking score calculations. No further modifications were applied to the default settings. The Glide protocol was initially tested for its ability to reproduce the crystallized binding mode of the N-terminal DWVI sequence. The program was successful in reproducing the experimentally determined binding mode of the tetrapeptide, as it corresponds to the best-scored pose of the 10 saved poses, thus validating the docking protocol.

The virtual screening of the fragment libraries (a total of about 34,000 3D structures) in the Trp2 E-cadherin pocket was performed by applying the same docking protocol and saving one pose for each fragment. Docking poses were filtered on the basis of their state penalty to exclude unfavorable tautomeric and protonation states (state penalty <0.6 kcal/mol), and then ranked according to the Glide docking score. The binding mode of the top 30 fragments of each library was carefully analyzed to identify the fragments that can fit into the Trp2 pocket and can form additional interactions within the pocket. In the re-docking step of the selected fragments, the Glide docking score was used to select 10 poses for each ligand (OPLS3 force field, SP scoring function).

### MD simulations

MD simulations were carried out using the software Desmond [Desmond (Schrödinger Release 2018-3)] in NVT conditions (T = 300 K, Langevin thermostat (Grest and Kremer, 1986) with relaxation time = 1.0 ps) considering the representative docking poses of fragments **1, 1a, 1b, 2, 3a, 4** and **5** as obtained from the analysis of the re-docking procedure. Each docking pose was solvated with a triclinic SPC (Berweger et al., 1995) box of 10 Å and neutralized by adding the proper number of Cl^−^ ions. The systems were equilibrated by applying the “desmond_nvt_relax.msj” protocol available in Desmond with the default parameters and the OPLS3e force field (Roos et al., 2019). Simulations of 500 ns were carried out saving 5,000 structures for the analysis.

We applied “Simulation interactions diagrams” and “Simulation Event analysis” tools of Desmond for the analysis of the trajectories and for the evaluation of the stability of the system. Protein stability was assessed by RMSDs (backbone atoms) and RMSFs (Cα atoms only) analysis.

### Cloning, expression, and purification of human E-cadherin-EC1-EC2

A DNA fragment encoding for the EC1-EC2 portion of human E-cadherin (residues 1-213) was cloned into a pET-3a expression vector (Novagen). The protein was fused at its N-terminus to a 6His-tag, a spacer peptide (Ser-Ser-Gly-His-Ile), and the enterokinase recognition site (Asp-Asp-Asp-Asp-Lys). The protein was expressed overnight at room temperature in BL21 (DE3)pLysS *E*. *coli* cells (Invitrogen). Cells were sonicated in TBS pH 7.4 + 1 mM CaCl_2_. The cell lysate was purified first by Ni-affinity chromatography and then by gel filtration using a Sephacryl 100 HR HiPrep 26/60 size exclusion column (GE Healthcare). The protein was then dialyzed in TBS pH 7.4 + 20 mM CaCl_2_, digested with enterokinase (New England Biolabs) at 25°C, and further purified by Ni-affinity chromatography. The flow-through fraction was collected and purified by size exclusion chromatography in TBS pH 7.4 + 1 mM CaCl_2_ for long-term storage.

### NMR studies

^1^H-NMR, 2D-COSY, and ^1^H, ^13^C-HSQC, and HMBC were used for fragment characterization and the assignments are reported in the Supplementary Tables S8–S14.

For STD spectra, all protein–ligand samples were prepared in a 50:1 ligand/protein ratio. Typically, in a solution made of 20 mM deuterated phosphate buffer pH 7.4, 150 mM NaCl and 1 mM CaCl_2,_ the final concentration of the ligand in the samples was 1 mM, while the final concentration of E-cadherin-EC1-EC2 was 20 μM. The final volume of the analyzed samples was 200 μl.

STD-NMR experiments were performed on a 600 MHz Bruker Avance spectrometer. The probe temperature was maintained at 298 K. In the STD experiments, water suppression was achieved by the WATERGATE 3-9-19 pulse sequence. The on-resonance irradiation of the protein was performed at −0.1 ppm. Off-resonance irradiation was applied at 200 ppm, where no protein signals are visible. Selective presaturation of the protein was achieved by a train of Gauss-shaped pulses of 49 ms in length each. The STD-NMR spectra were acquired with an optimized total length of saturation train of 0.98 and 2.94 s. Blank experiments were conducted in the absence of protein in order to avoid artifacts. STD experiments were integrated and the effect is expressed as (I0−Isat)/I0, expressing the signal intensity in the STD spectrum as a fraction of the intensity of an unsaturated reference spectrum. In this equation, I0 is the intensity of one signal in the off-resonance or reference NMR spectrum, Isat is the intensity of a signal in the on-resonance NMR spectrum, and I0–Isat represents the intensity of the STD-NMR spectrum. Further processing for building epitope maps within single ligands involved the calculation of two related values, absolute and relative STD intensities (both values are given as percentages). To facilitate comparison of protons within a single molecule, relative STD % was subsequently calculated: the proton with the highest absolute STD % was given the arbitrary value of 100%; the values of the other protons were then calculated relative to this proton.

The relative STD % values obtained from the spectra were used for epitope mapping representation (Figure 4).

The absolute STD values obtained for the aromatic and aliphatic protons of each fragment are reported in Supplementary Table S15, showing the data obtained with two different irradiation times (0.98 and 2.94 s). Since significantly different T1 relaxation times of the ligand protons (particularly when they have different features, such as aromatic and aliphatic hydrogens) can produce artifacts in the epitope definition, these two irradiation times were considered in order to demonstrate that epitope information depends neither on the chosen saturation time nor on the ability to accumulate saturation in the free state. Considering that the effects observed in the two STD spectra are comparable, the data obtained at 2.94 s are discussed in the results section.

## Data Availability

The original contributions presented in the study are included in the article/Supplementary Material; further inquiries can be directed to the corresponding author.
