# Supplementary material for: A combined fragment-based virtual screening and STD-NMR approach for the identification of E-cadherin ligands
Source: Front Chem. 2022 Aug 19;10:946087. doi: 10.3389/fchem.2022.946087 (PMC9437437; doi:10.3389/fchem.2022.946087)
Supplement: Supplementary file 1 [file DataSheet1.docx]

Supplementary Material

**Re-docking of the selected fragments 2**

**Molecular dynamics results 3**

**NMR assignments and spectra 11**

**Chemical synthesis of fragments 1b and 1a 23**

**Re-docking of the selected fragments**

**Fragment 1**: The analysis of the docking poses shows the two binding modes A and B, differing in the orientation of the aromatic moiety within the Trp2 pocket. The type A, which includes the best pose and represents the most populated binding mode, is characterized by the classical hydrogen bond between the indole NH and the backbone C=O of Asp90; in type B the indole in the pocket is oriented to form a hydrogen bond with the backbone C=O of Lys25. All the poses engage both or one of the cyclic amines in salt bridges with residues of the pocket (Glu89, Asp90) or the adhesion arm (Asp1). In the two less favored poses the indole ring is out of the cavity.

**Fragments 1a**: The enantiomer *S*-**1a** shows only type A poses, while *R*-**1a** prefers type A with a minor contribution of type B. The charged amines form salt bridges with the residues of the pocket, i.e. Glu89 or Asp90, and of the adhesive arm (Asp1). The best pose (type A) of both enantiomers was selected for MD simulations.

**Fragments 1b**: In the best poses, both enantiomers insert the indole ring in the Trp2 pocket, with *R*-**1b** forming a hydrogen bond with the backbone of Asp90 (type A) and *S*-**1b** with the backbone of Lys25 (type B). The binding mode of type A is preferred by *R*-**1b** while *S*-**1b** alternates both types. In the best pose both fragments form a salt bridge between the cyclic amine and the Glu89 side chain while the amide group is oriented between the side chain of Asp90 and Asp1 and towards Glu89 for *R*-**1b** and *S*-**1b**, respectively. For MD simulations, we selected the best pose (type A) of *R*-**1b** and the best pose (type B) and the second-ranked pose (type A) of *S*-**1b**.

**Fragment 2**. The top-ranked pose corresponds to the binding mode B which alternates with type A among the poses. The charged amine shows a preferred interaction with the adhesive arm residues, forming a salt bridge with the side chain of Asp1 or a hydrogen bond with the backbone C=O of Trp2. Both binding modes were selected for MD simulations.

**Fragment 3a**. The most energetically favored binding mode is similar to type B. In the best pose the fragment displays a hydrogen bond with the backbone of Lys25 and forms salt bridges with the side chain of Glu89, Asp90 and Asp1. Among the saved poses, the aniline ring also forms hydrogen bond with the backbone of Asp90 recapturing the type A pose. Both binding modes form salt bridges with the side chain of Glu89, Asp90 or Asp1. The type A and B poses were selected for MD simulations.

**Fragment 4**. All the poses are similar to type A with the amine forming a salt bridge with Asp1 side chain.

**Fragment 5**. The top-ranked pose corresponds to the type A which alternates with type B maintaining a preferred salt bridge between the amine and the side chain of Glu89.

**Table S1**. Percentages of salt bridges formed by *S*-**1a** and *R*-**1a** amine groups with the protein residues in MD simulations.

| Fragment -Residue | *S***-1a**, pose A | | *R*-**1a**, pose A | |
| --- | --- | --- | --- | --- |
|  | %* | Average and  std values (Å) | %* | Average and  std values (Å) |
| NH_3_^+^ter-Asp1 | 65 | 4.75±1.77 | 0 | 12.25±2.67 |
| NH_3_^+^ter-Glu89 | 0 | 6.96±0.72 | 64 | 4.52±1.22 |
| NH_3_^+^ter-Asp90 | 74 | 4.05±1.24 | 92 | 3.77±0.97 |
| NH_2_^+^-Asp1 | 69 | 4.36±1.36 | 0 | 11.29±2.09 |
| NH_2_^+^-Glu89 | 93 | 3.35±0.60 | 32 | 5.27±1.12 |
| NH_2_^+^-Asp90 | 0 | 6.35±1.00 | 0 | 6.97±1.02 |

* distance between fragment N and cadherin side chain carbon COO- ≤ 5Å

**Table S2**. Percentages of salt bridges formed by *S*-**1b** and *R*-**1b** amine groups with the protein residues in MD simulations.

| Fragment -Residue | *S***-1b** | | | | | *R***-1b**, pose A | |
| --- | --- | --- | --- | --- | --- | --- | --- |
|  | %* | | | Average and  std values (Å) | | %* | Average and  std values (Å) |
|  | Pose A | Pose B | | Pose A | Pose B |  |  |
| NH_2_^+^-Asp1 | 85 | | 60 | 3.98±1.16 | 4.82 ±1.74 | 72 | 4.44±1.51 |
| NH_2_^+^-Glu89 | 93 | | 47 | 3.87±0.69 | 4.94±1.00 | 87 | 3.88±0.68 |
| NH_2_^+^-Asp90 | 0 | | 0 | 7.17±0.74 | 8.13±1.26 | 0 | 7.61±0.93 |

* distance between fragment N and cadherin side chain carbon COO- ≤ 5

**Table S3**. Percentages of salt bridges formed by **1** amine groups with the protein residues in MD simulations.

| Fragment -Residue | Pose A | | Pose B | |
| --- | --- | --- | --- | --- |
|  | %* | Average and  std values (Å) | %* | Average and  std values (Å) |
| NH_2_^+^ter-Asp1 | 0 | 8.91±2.99 | 60 | 4.48±1.24 |
| NH_2_^+^ter-Glu89 | 10 | 6.20±0.53 | 0 | 6.97±0.63 |
| NH_2_^+^ter-Asp90 | 83 | 3.85±1.01 | 0 | 11.55±1.24 |
| NH_2_^+^-Asp1 | 26 | 6.85±2.50 | 52 | 5.12±1.72 |
| NH_2_^+^-Glu89 | 21 | 5.37±0.91 | 67 | 4.48±0.78 |
| NH_2_^+^-Asp90 | 0 | 6.41±0.57 | 4 | 7.47±1.32 |

* distance between fragment N and cadherin side chain carbon COO- ≤ 5Å

**Molecular dynamics results for fragments 2-5**

**Fragment 2.** Both binding modes keep the indole ring inside the hydrophobic pocket conserving the hydrogen bond with Asp90 (pose A, 90% populated) or Lys 25 (pose B, 77% populated). The binding mode A is further stabilized by a salt bridge with Asp1 side chain (51%, Table S4) and a hydrogen bond between the amine of the piperidine ring and the carbonyl group of Trp2 backbone (21% populated, distance between fragment N and Trp2 carbonyl O ≤ 4Å) which are formed and broken during the simulation (Supplementary Figure 1). Similar results are observed for the pose B which, as observed for A, prefers to interact with the adhesive arm residues (the hydrogen bond with Trp2 is 39% populated)


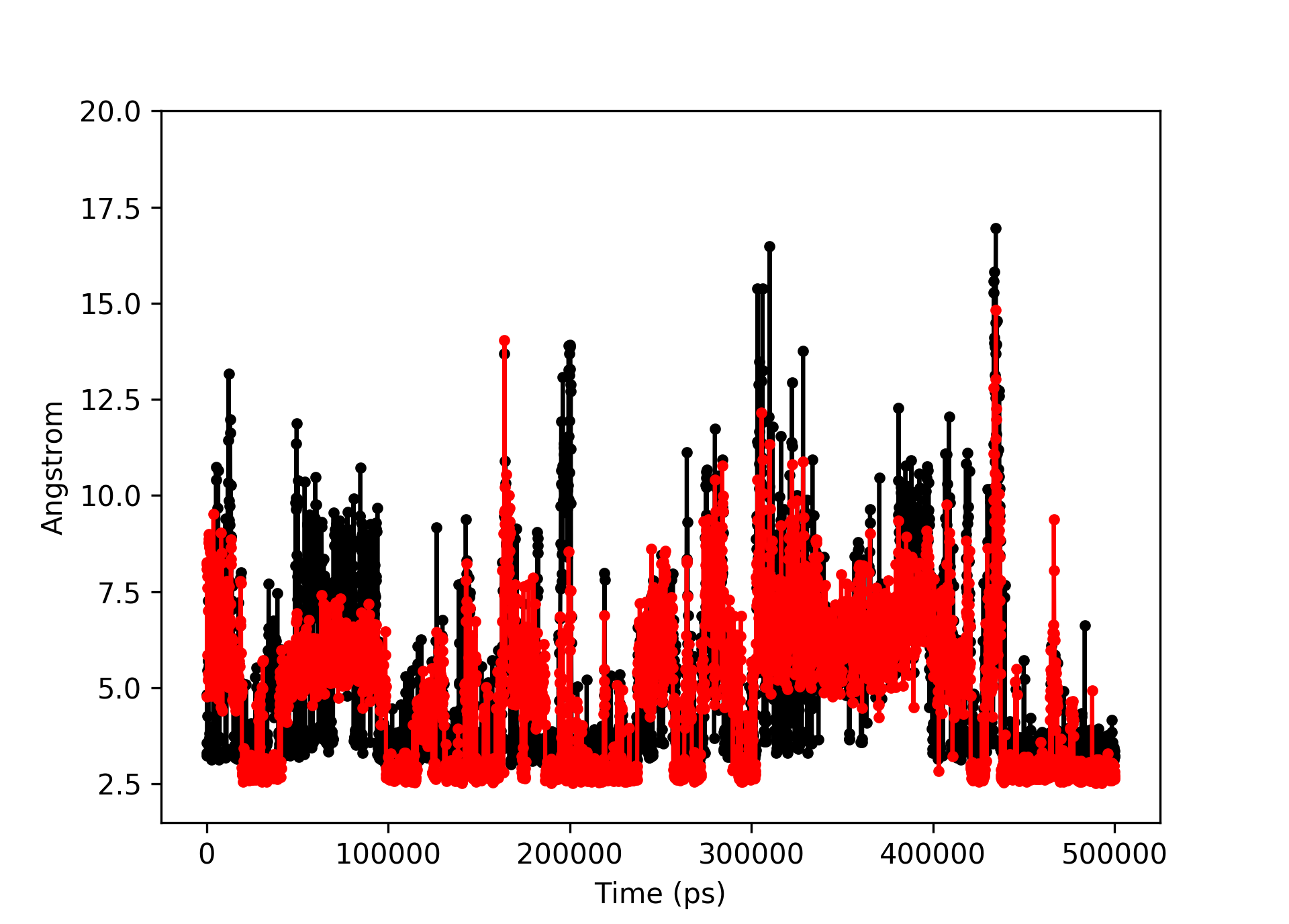

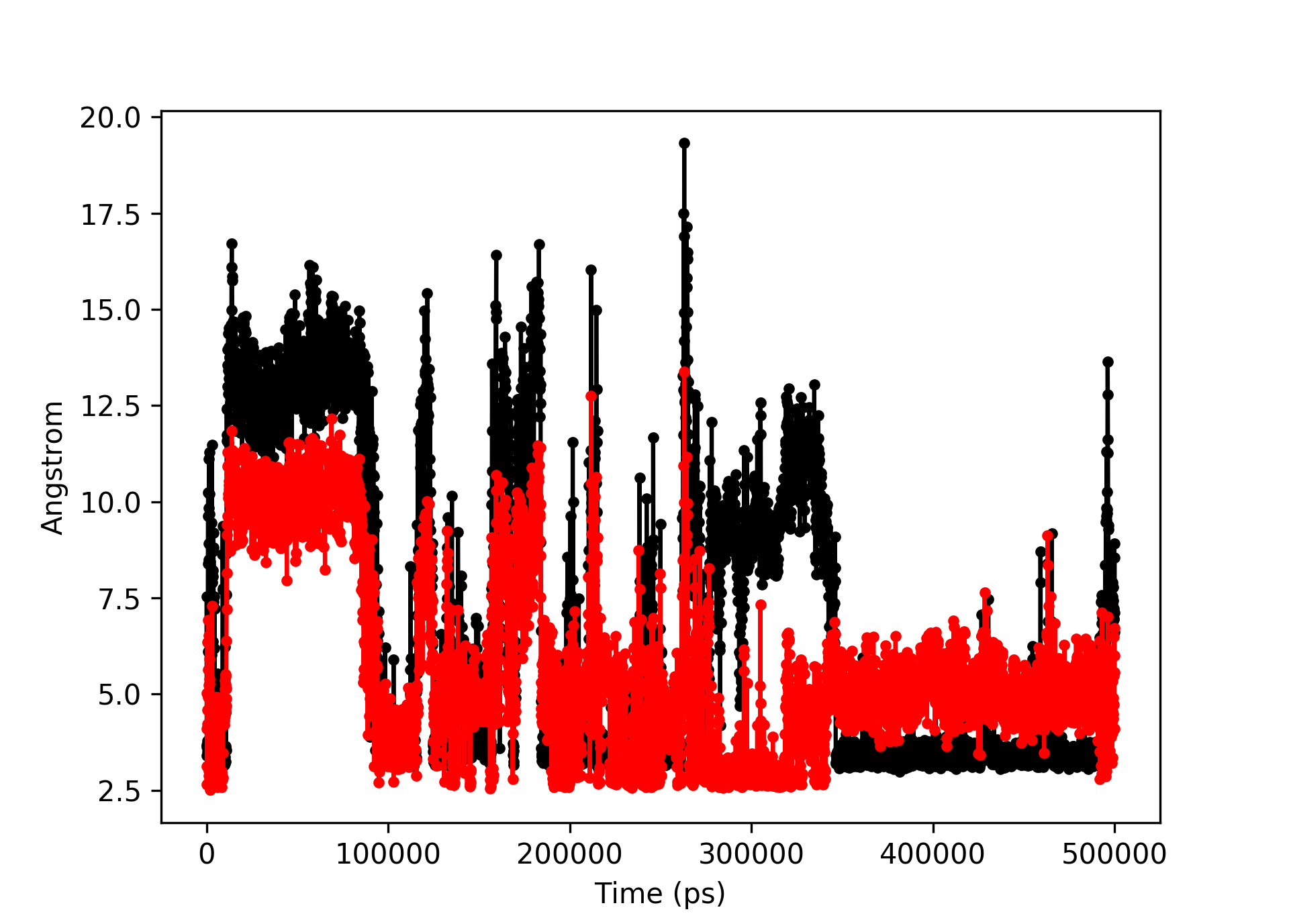


**Supplementary Figure 1.** Plots (left, pose A: right, pose B) of the distances between the fragment piperidine nitrogen and Asp1 carbon of COO- group (black) and the oxygen of the Trp2 carbonyl group (red).

**Table S4**. Percentage of salt bridges formed by the amine group of fragment **2** with the protein residues.

| Fragment -Residue | Pose A | | Pose B | |
| --- | --- | --- | --- | --- |
|  | %* | Average and  std values (Å) | %* | Average and  std values (Å) |
| NH_2_^+^-Asp1 | 51 | 6.86±3.97 | 59 | 5.04±2.18 |
| NH_2_^+^-Glu89 | 15 | 6.37±1.17 | 15 | 6.05±1.08 |
| NH_2_^+^-Asp90 | 0 | 9.98±1.23 | 5 | 6.87±1.37 |

* distance between fragment N and cadherin side chain carbon COO- ≤ 5Å

*Comparison with STD-NMR data*. In the STD-NMR spectrum of fragment **2** we observed both the aromatic and aliphatic protons (the latter showing weaker interaction than the aromatic ones). Indeed, the indole ring remains bound to the Trp2 pocket and the piperidine ring forms interactions with the adhesive arm residues Asp1 and Trp2.

**Fragment 3a.** During the simulation starting from pose B the hydrogen bond between the aniline ring and the backbone of Lys25 is replaced by a stable hydrogen bond with the backbone of Ile24 (94% populated) and an additional hydrogen bond with the side chain of Ser78 (68% populated) is formed (Supplementary Figure 2). The salt bridge between the cyclic amine and the side chain of Glu89 is stable while the salt bridge between the terminal amine and the side chain of Asp1 is formed and broken during the simulation (Table S5). The type A pose is less stable and during the simulation the fragment leaves the binding site.


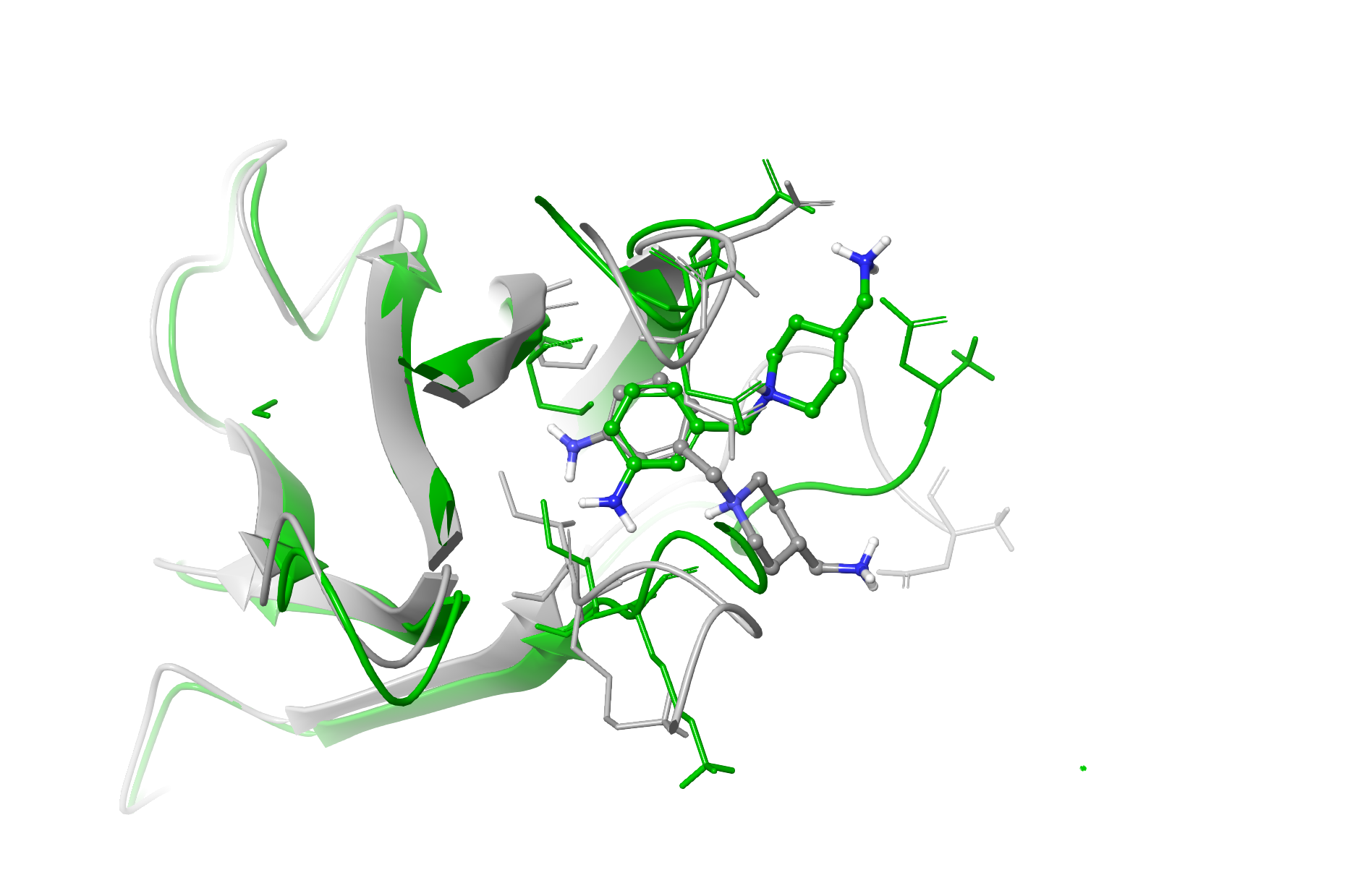


D1

D1

D90

K25

S78

I24

E89

**Supplementary Figure 2**. Snapshot taken form MD trajectory of **3a** (grey) overlaid to the starting geometry (green). The key interactions observed during the simulation with the protein residues are shown.

**Table S5**. Percentage of salt bridges formed by the amine groups of fragment **3a** with the protein residues.

| Fragment -Residue | Pose B | |
| --- | --- | --- |
|  | %* | Average and  std values (Å) |
| NH_3_^+^ter-Asp1 | 38 | 10.95±6.87 |
| NH_3_^+^ter-Glu89 | 0 | 9.01±0.97 |
| NH_3_^+^ter-Asp90 | 3 | 10.55±2.90 |
| NH^+^-Asp1 | 0 | 14.65±6.24 |
| NH^+^-Glu89 | 68 | 4.81±0.43 |
| NH^+^-Asp90 | 0 | 8.69±1.37 |

* distance between fragment N and side chain carbon COO- ≤ 5Å

*Comparison with STD-NMR data*. There is a good agreement with NMR data, showing high STD% for the aromatic protons and low STD% for the methylene group of methylamine. Indeed the aniline ring is stable inside the pocket and the terminal amine can form ionic-ionic interaction with Asp1.

**Fragment 4.** The salt bridge with Asp1 and the hydrogen bond with Asp90, formed in the starting docking pose, are lost after about 280ns (34% and 28% populated, respectively, Supplementary Figure 3) and only the salt bridge with Glu89 is conserved (Table S6). However, even if the fragment remains bound to the hydrophobic pocket, it changes its orientation lacking the stabilizing hydrogen bond with Asp90 (Supplementary Figure 4).


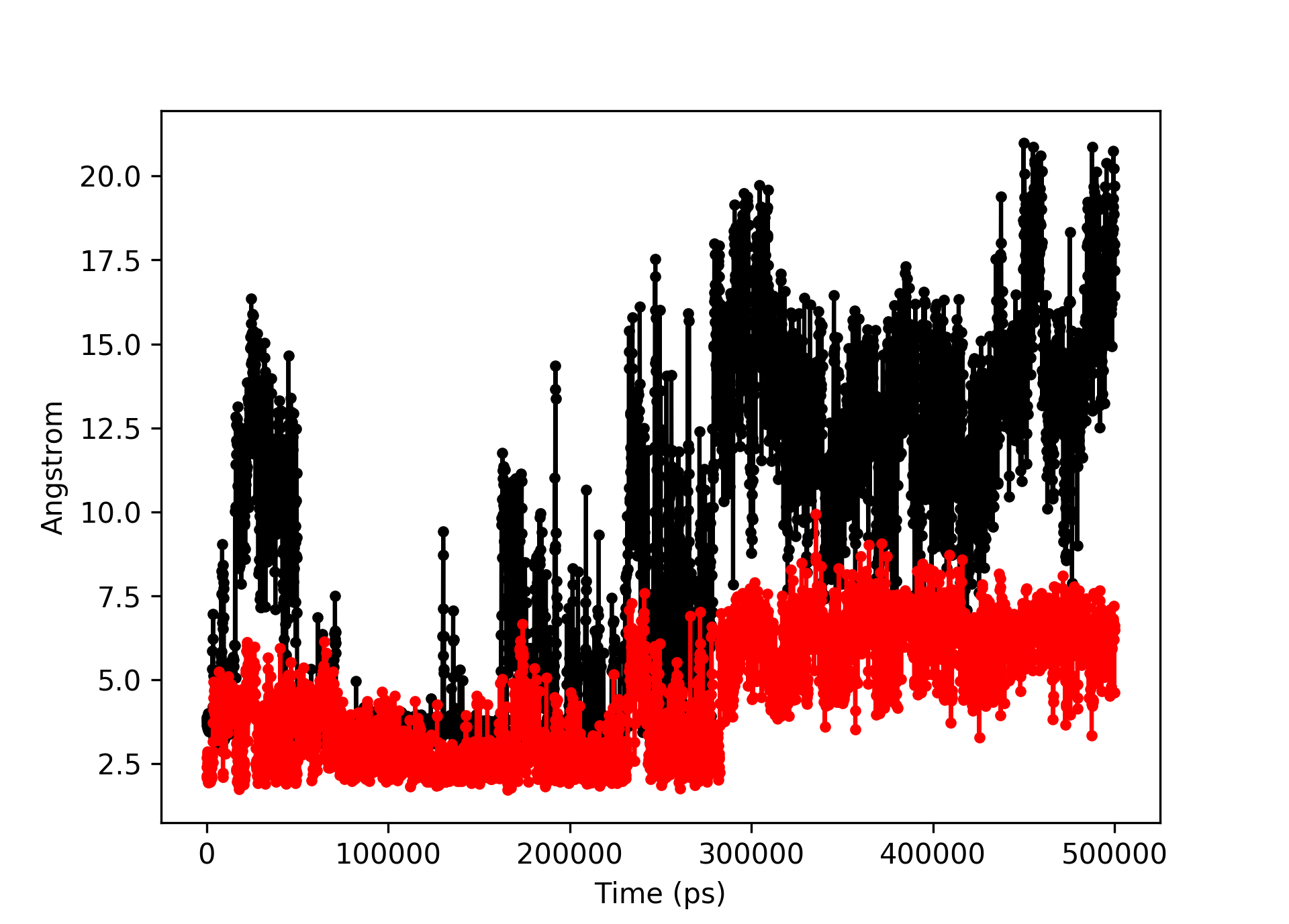


**Supplementary Figure 3.** Plots of the distances between the fragment **4** neutral nitrogen and Asp90 oxygen of C=O group (red) and between the fragment charged nitrogen and Asp1 carbon of COO- group (black).

**Table S6**. Percentage of salt bridges formed by amine group of fragment **4** with the protein residues.

| Fragment -Residue | Pose A | |
| --- | --- | --- |
|  | %* | Average and  std values (Å) |
| NH_3_^+^-Asp1 | 34 | 9.13±4.89 |
| NH_3_^+^-Glu89 | 82 | 4.14±1.19 |
| NH_3_^+^-Asp90 | 35 | 6.31±2.26 |

* distance between fragment N and side chain carbon COO- ≤ 5Å


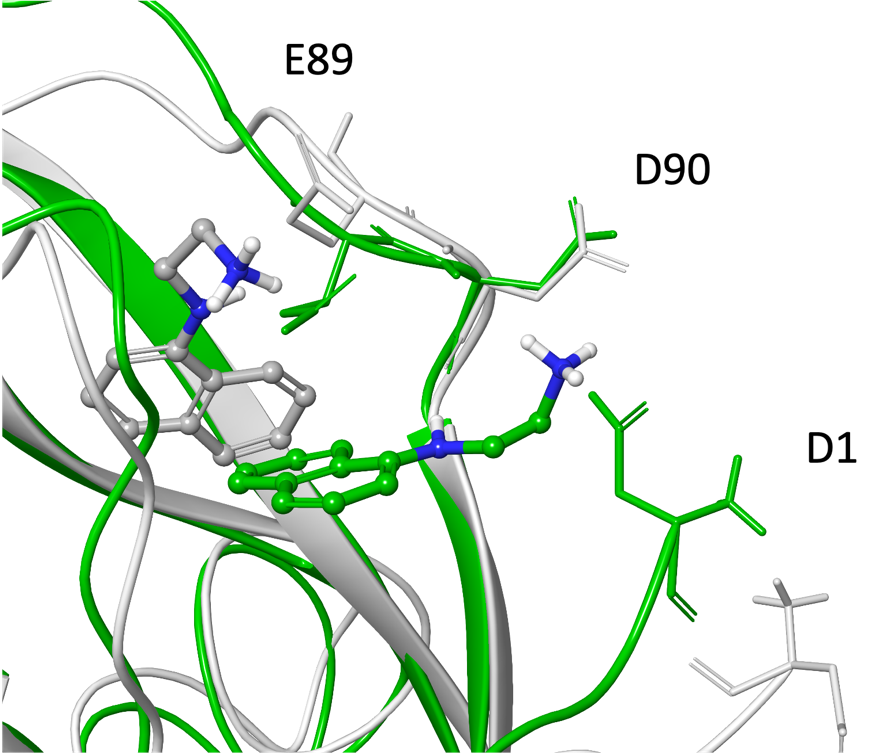


**Supplementary Figure 4**. Superimposition of the first (green) and last (grey) MD simulation frames of fragment **4**.

*Comparison with STD-NMR data*. Only the aromatic protons contribute to the STD-NMR spectrum. Indeed fragment **4** reveals a less stable binding mode with the Trp2 pocket, losing the hydrogen bond with Asp90 and the salt bridge with Asp1 and showing great mobility inside the pocket. For this fragment, the lacking of a stable hydrogen bond donor group on the aromatic ring prevents an efficient interaction.

**Fragment 5.** The benzimidazole ring is stable in the hydrophobic pocket and always forms the hydrogen bond with Asp90. The charged amino group is sandwiched between the side chain of Glu89 and Asp1 (Table S7).

**Table S7**. Percentage of salt bridges formed by amine group of fragment **5** with the protein residues.

| Fragment -Residue | Pose A | |
| --- | --- | --- |
|  | %* | Average and  std values (Å) |
| NH_3_^+^-Asp1 | 84 | 4.39±2.31 |
| NH_3_^+^-Glu89 | 97 | 3.62±0.43 |
| NH_3_^+^-Asp90 | 0 | 6.87±0.93 |

* distance between fragment N and side chain carbon COO- ≤ 5Å

*Comparison with STD-NMR data*. Besides the aromatic protons, also the aliphatic protons contribute to the spectrum. According to MD results, the benzimidazole ring is indeed tightly bound to the hydrophobic pocket and the charged amine is stabilized by both Asp1 and Glu89 residues.

**NMR assignments and spectra**

**Table S8**. ^1^H and ^13^C NMR assignment for fragment **1** in deuterated phosphate buffer.

| 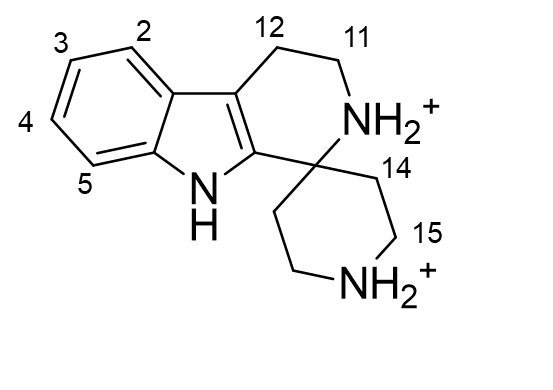 | | | |
| --- | --- | --- | --- |
| **Atom** | **δ ^1^H (ppm)** | **m** | **δ ^13^C (ppm)** |
| **2** | 7,5172 | d (J=8) | 118,32 |
| **3** | 7,0852 | t (J=8) | 119,45 |
| **4** | 7,1718 | t (J=8) | 122,12 |
| **5** | 7,3972 | d (J=8) | 111,44 |
| **11** | 3,037 | t (J=7) | 38,8 |
| **12** | 2,693 | t (J=7) | 21,3 |
| **15** | 3,274 | m | 39,1 |
| **14** | 2,15 - 1,91 | m | 31,5 |

**Table S9**. ^1^H and ^13^C NMR assignment for fragment **1a** in deuterated phosphate buffer.

| 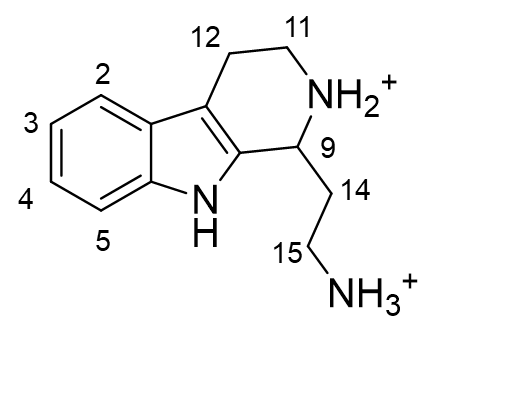 | | | |
| --- | --- | --- | --- |
| **Atom** | **δ ^1^H (ppm)** | **m** | **δ ^13^C (ppm)** |
| **2** | 7,54 | d (J=8) | 118,3 |
| **3** | 7,11 | t (J=8) | 120 |
| **4** | 7,20 | t (J=8) | 122,6 |
| **5** | 7,42 | d (J=8) | 111,7 |
| **9** | 4,7 | s | 50,7 |
| **11** | 3,59 - 3,36 | m | 40,9 |
| **12** | 2,97 | m | 18,5 |
| **14** | 2,47 – 2,3 | m | 29,5 |
| **15** | 3,18 | m | 35,8 |

**Table S10**. ^1^H and ^13^C NMR assignment for fragment **1b** in deuterated phosphate buffer.

| 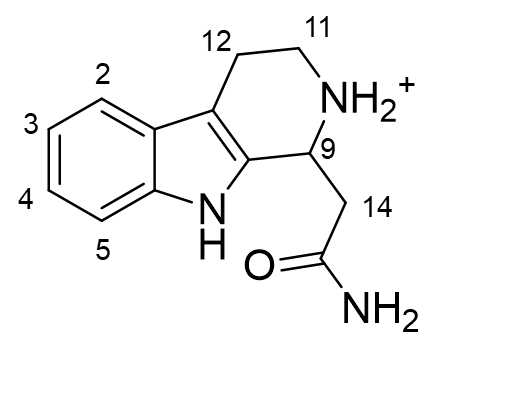 | | | |
| --- | --- | --- | --- |
| **Atom** | **δ ^1^H (ppm)** | **m** | **δ ^13^C (ppm)** |
| **2** | 7,54 | d (J=8) | .118,4 |
| **3** | 7,12 | t (J=8) | 119,9 |
| **4** | 7,22 | t (J=8) | 122,9 |
| **5** | 7,42 | d (J=8) | 111,7 |
| **9** | 5,0 | / | 50,0 |
| **11** | 3,66-3,41 | m | 41,5 |
| **12** | 3,02 | m | 18,0 |
| **14** | 3,12  2,97 | dd (J=4,5 - 16,9)  dd (J=7,5 - 16,9) | 35,5 |

**Table S11**. ^1^H and ^13^C NMR assignment for fragment **2** in deuterated phosphate buffer.

| 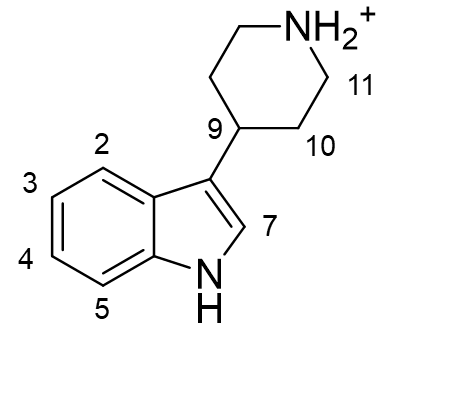 | | | |
| --- | --- | --- | --- |
| **Atom** | **δ ^1^H (ppm)** | **m** | **δ ^13^C (ppm)** |
| **2** | 7,64 | d (J=8) | 118,7 |
| **3** | 7,08 | t (J=8) | 119,0 |
| **4** | 7,17 | t (J=8) | 122,0 |
| **5** | 7,43 | d (J=8) | 111,9 |
| **7** | 7,16 | s | 121,3 |
| **9** | 3,15 | m | 30,6 |
| **10** | 2,21 – 1,87 | m | 29,1 |
| **11** | 3,45 – 3,14 | m | 44,3 |

**Table S12**. ^1^H and ^13^C NMR assignment for fragment **3a** in deuterated phosphate buffer.

| **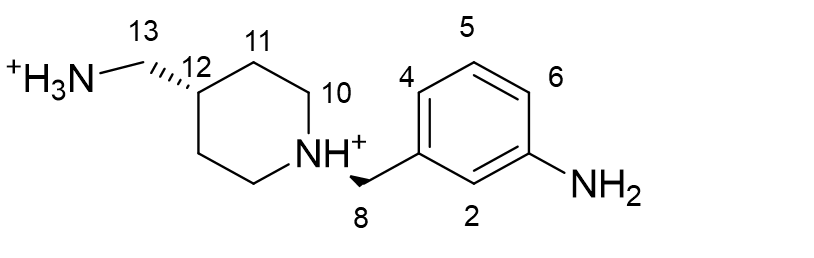** | | | |
| --- | --- | --- | --- |
| **Atom** | **δ ^1^H (ppm)** | **m** | **δ ^13^C (ppm)** |
| **2** | 6,75 | m | 117,9 |
| **4** | 6,77 | m | 121,0 |
| **5** | 7,19 | t (J=8) | 129,4 |
| **6** | 6,77 | m | 115,2 |
| **8** | 4.01 | bs | 61,7 |
| **10** | 3,36 – 2,82 | m | 52,1 |
| **11** | 1,91 -1,42 | m | 27,5 |
| **13** | 2,87 | d (J=7) | 44,6 |

**Table S13**. ^1^H and ^13^C NMR assignment for fragment **4** in deuterated phosphate buffer.

| 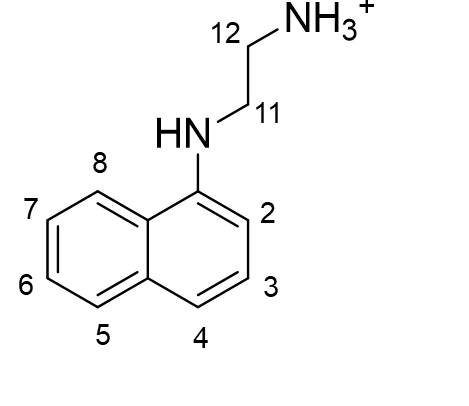 | | | |
| --- | --- | --- | --- |
| **Atom** | **δ ^1^H (ppm)** | **m** | **δ ^13^C (ppm)** |
| **2** | 6,82 | dd (J=8, 4) | 106,5 |
| **3** | 7,41 | m | 126,5 |
| **4** | 7,41 | m | 119,5 |
| **5** | 7,86 | m | 128,5 |
| **6** | 7,52 | m | 126,2 |
| **7** | 7,52 | m | 126,2 |
| **8** | 7,97 | m | 120,5 |
| **11** | 3,63 | t (J=7) | 41,1 |
| **12** | 3,31 | t (J=7) | 37,8 |

**Table S14**. ^1^H and ^13^C NMR assignment for fragment **5** in deuterated phosphate buffer.

| 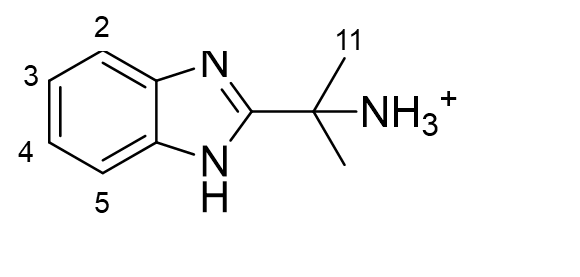 | | | |
| --- | --- | --- | --- |
| **Atom** | **δ ^1^H (ppm)** | **m** | **δ ^13^C (ppm)** |
| **2, 5** | 7,61 | m | 115,2 |
| **3, 4** | 7,30 | m | 123,4 |
| **11** | 1,79 | s | 25,3 |


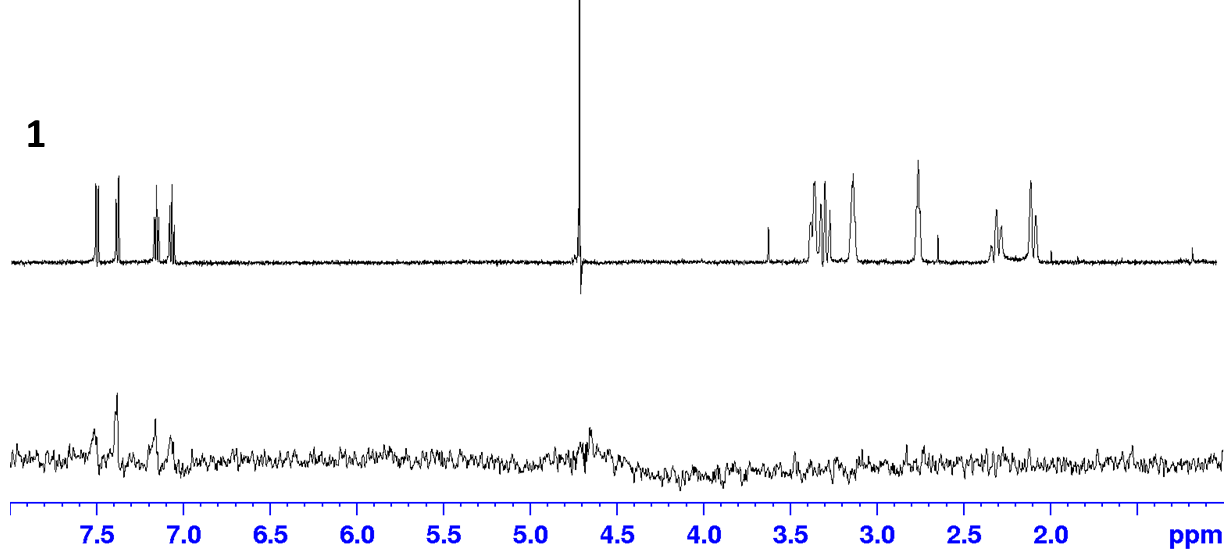


**Supplementary Figure 5**. ^1^H-NMR (top) and STD-NMR (bottom) spectra of fragment **1** in presence of E-cadherin. Spectra were acquired with a Bruker Avance 600 MHz spectrometer at T=298K. STD spectrum is acquired irradiating at -0.1 ppm and with a saturation time of 2.94s.


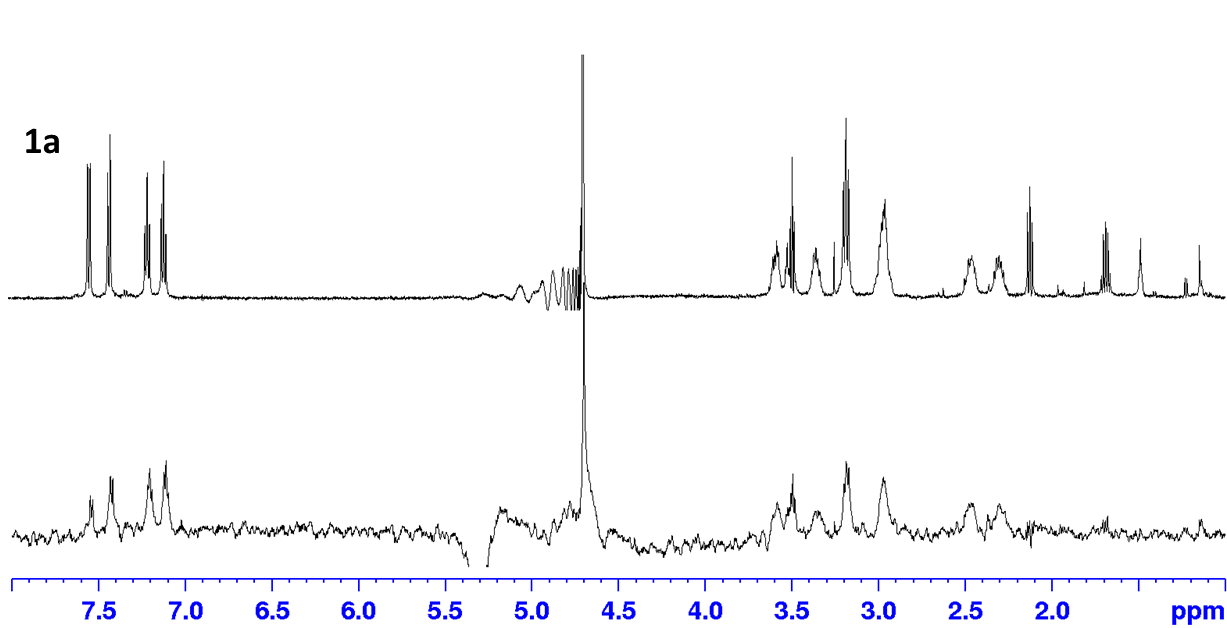


**Supplementary Figure 6**. ^1^H-NMR (top) and STD-NMR (bottom) spectra of fragment **1a** in presence of E-cadherin. Spectra were acquired with a Bruker Avance 600 MHz spectrometer at T=298K. STD spectrum is acquired irradiating at -0.1 ppm and with a saturation time of 2.94s.


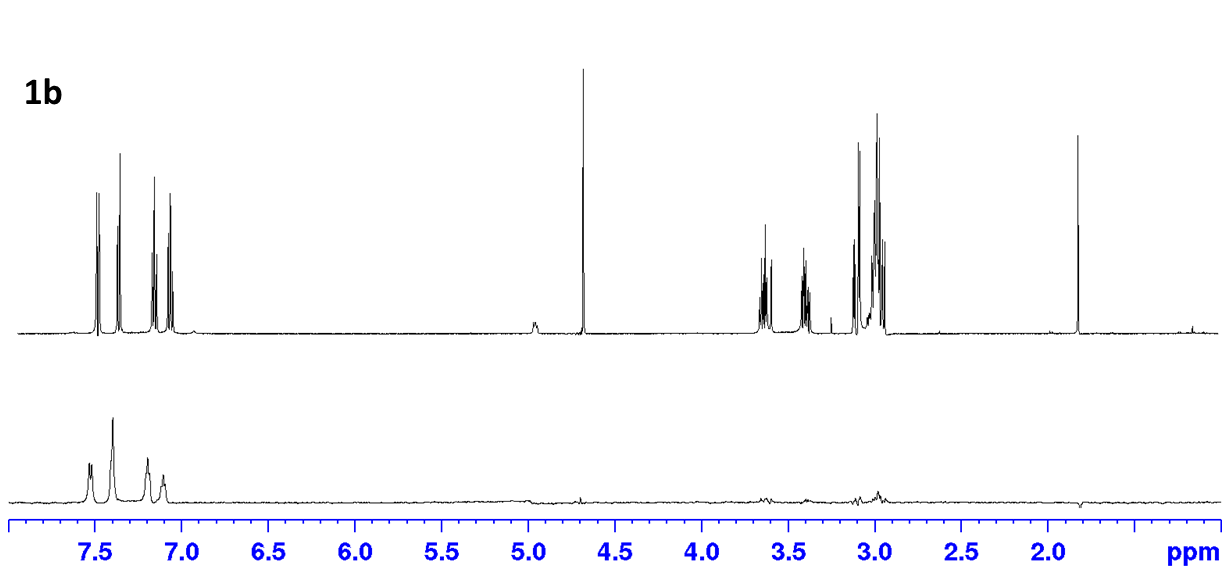


**Supplementary Figure 7**. ^1^H-NMR (top) and STD-NMR (bottom) spectra of fragment **1b** in presence of E-cadherin. Spectra were acquired with a Bruker Avance 600 MHz spectrometer at T=298K. STD spectrum is acquired irradiating at -0.1 ppm and with a saturation time of 2.94s.


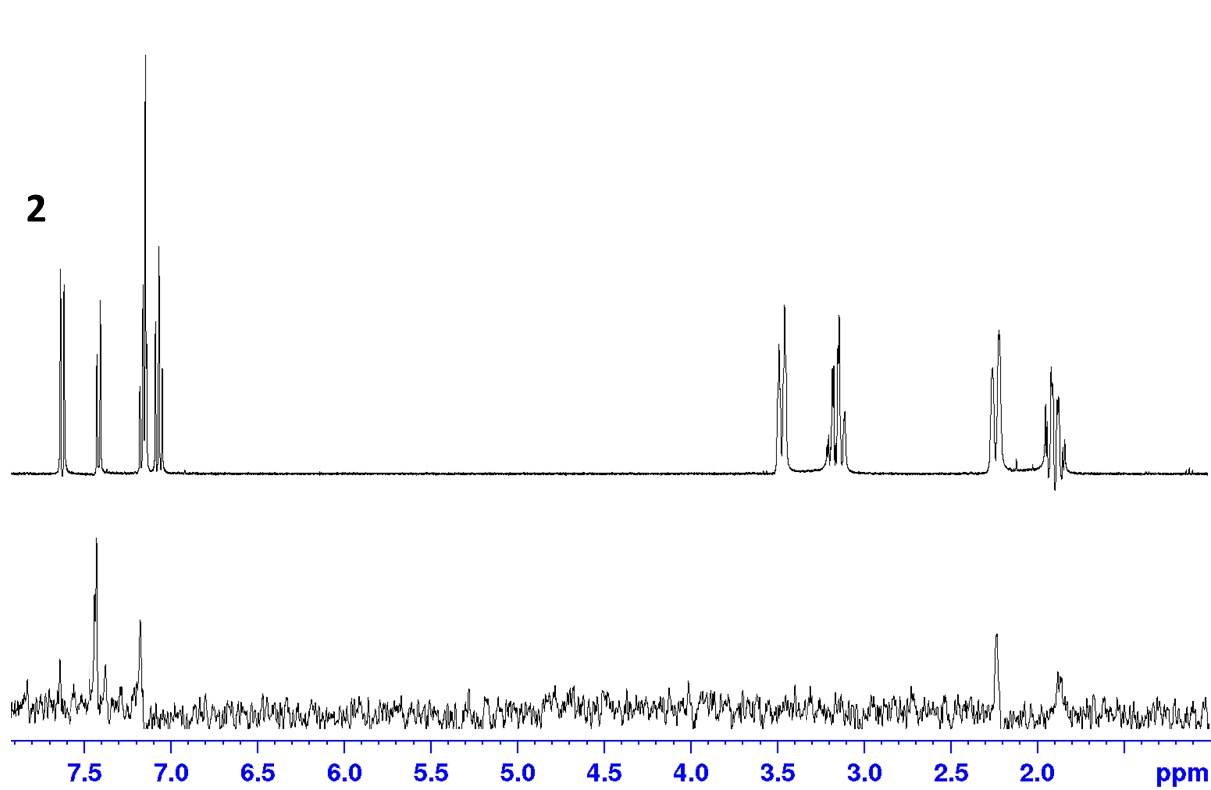


**Supplementary Figure 8**. ^1^H-NMR (top) and STD-NMR (bottom) spectra of fragment **2** in presence of E-cadherin. Spectra were acquired with a Bruker Avance 600 MHz spectrometer at T=298K. STD spectrum is acquired irradiating at -0.1 ppm and with a saturation time of 2.94s.


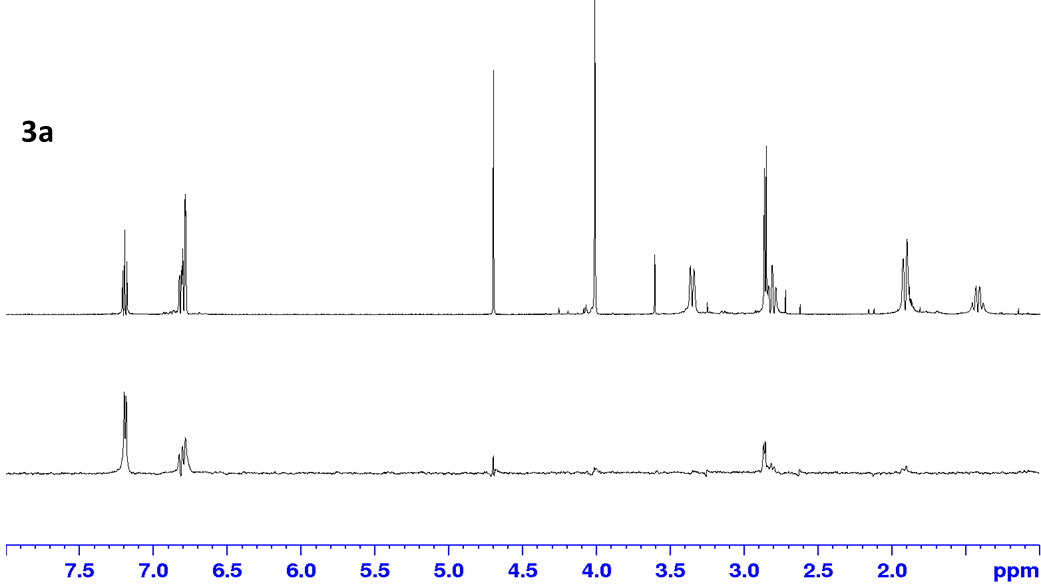


**Supplementary Figure 9**. ^1^H-NMR (top) and STD-NMR (bottom) spectra of fragment **3a** in presence of E-cadherin. Spectra were acquired with a Bruker Avance 600 MHz spectrometer at T=298K. STD spectrum is acquired irradiating at -0.1 ppm and with a saturation time of 2.94s.


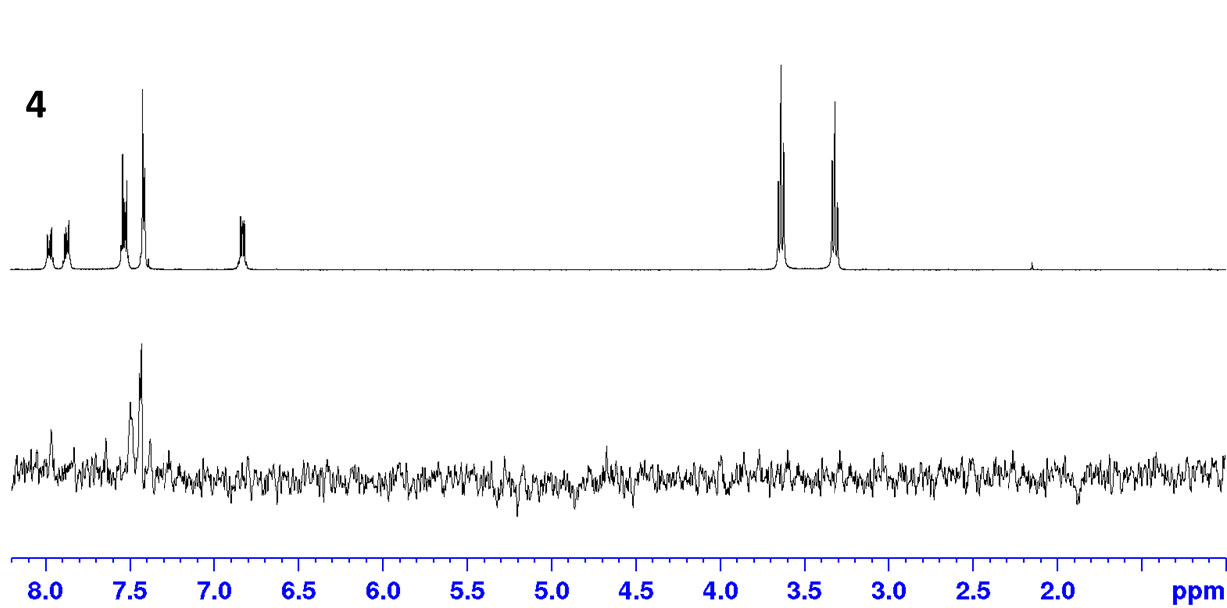


**Supplementary Figure 10**. ^1^H-NMR (top) and STD-NMR (bottom) spectra of fragment **4** in presence of E-cadherin. Spectra were acquired with a Bruker Avance 600 MHz spectrometer at T=298K. STD spectrum is acquired irradiating at -0.1 ppm and with a saturation time of 2.94s.


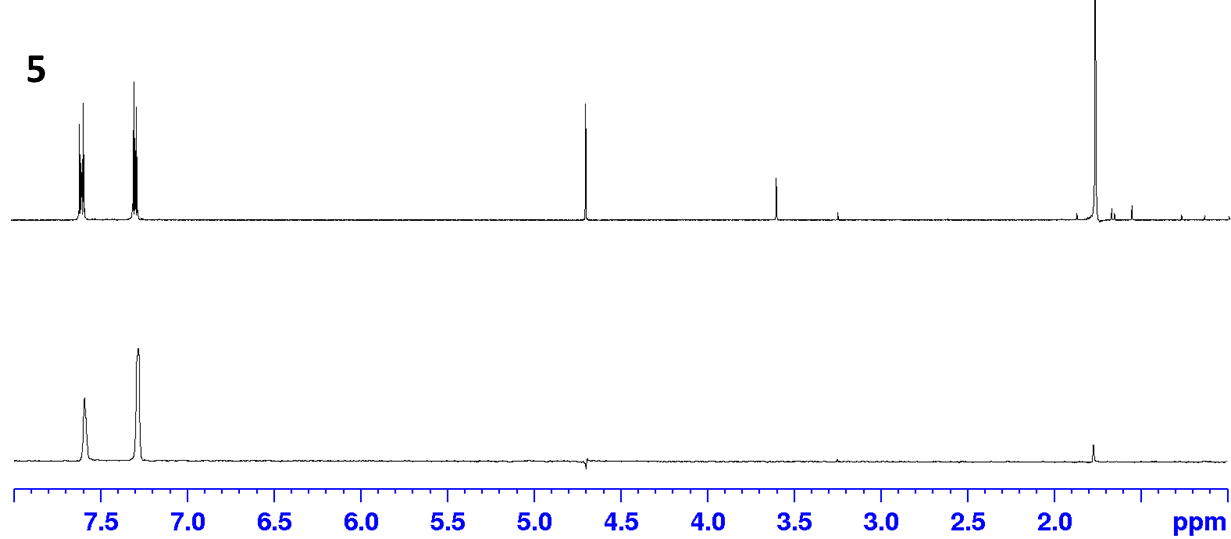


**Supplementary Figure** **11**. ^1^H-NMR (top) and STD-NMR (bottom) spectra of fragment **5** in presence of E-cadherin. Spectra were acquired with a Bruker Avance 600 MHz spectrometer at T=298K. STD spectrum is acquired irradiating at -0.1 ppm and with a saturation time of 2.94s.

**Table S15**. STD absolute % for aromatic and aliphatic protons of each fragment in presence of E-cadherin obtained with a saturation time of 0.98 and 2.94 s, respectively.

|  |  | **T_sat_ = 0.98** | **T_sat_ = 2.94** |
| --- | --- | --- | --- |
| **1** | **Aromatic** | 1.2 | 2 |
|  | **Aliphatic** | 0.06 | / |
| **1a** | **Aromatic** | 3.7 | 6.2 |
|  | **Aliphatic** | 2.1 | 3.6 |
| **1b** | **Aromatic** | 1.0 | 1.5 |
|  | **Aliphatic** | 0.16 | 0.18 |
| **2** | **Aromatic** | 2.2 | 3.1 |
|  | **Aliphatic** | 0.8 | 1 |
| **3** | **Aromatic** | 0.52 | 0.78 |
|  | **Aliphatic** | 0.11 | 0.2 |
| **4** | **Aromatic** | 1.9 | 3.1 |
|  | **Aliphatic** | 0.06 | / |
| **5** | **Aromatic** | 2.58 | 3.1 |
|  | **Aliphatic** | 0.54 | 0.6 |

**Chemical synthesis of fragment 1b**

To a solution of tryptamine (500 mg, 3.12 mmol) in 6.25 mL of glacial acetic acid were added 314.8 mg (3.74 mmol) of cyanoacetamide and 65.62 mg of 10% Pd/C. The mixture was stirred at room temperature for 48 hours and checked by LC-MS analysis. The reaction was then neutralized using a 25% aqueous solution of ammonia, and the resulted mixture was filtered on celite pad to remove the catalyst and washed with 50 mL of CH_2_Cl_2_. The solution was extracted with CH_2_Cl_2_ (3x20 mL), and the combined organic phases were dried over Na_2_SO_4_, filtered and concentrated under reduced pressure. The crude product was purified by silica gel chromatography (eluent: AcOEt/MeOH 80:20) affording a yellow solid (51% yield).

^1^H NMR (400 MHz, MeOD) δ 7.36 (d, *J* = 9.6 Hz, 1H), 7.25 (d, J = 9.8 Hz, 1H), 7.02 (t, *J* = 7.6 Hz, 1H), 6.94 (t, J = 7.4 Hz, 1H), 4.53 (m, 1H), 3.33-3.26 (m, 2H), 3.12-2.99 (m, 1H), 2.87 (dd, *J*=15.9, 4.5, 1H), 2.80-2.71 (m, 2H), 2.65 (dd, *J*= 15.9, 8.8 Hz, 1H).


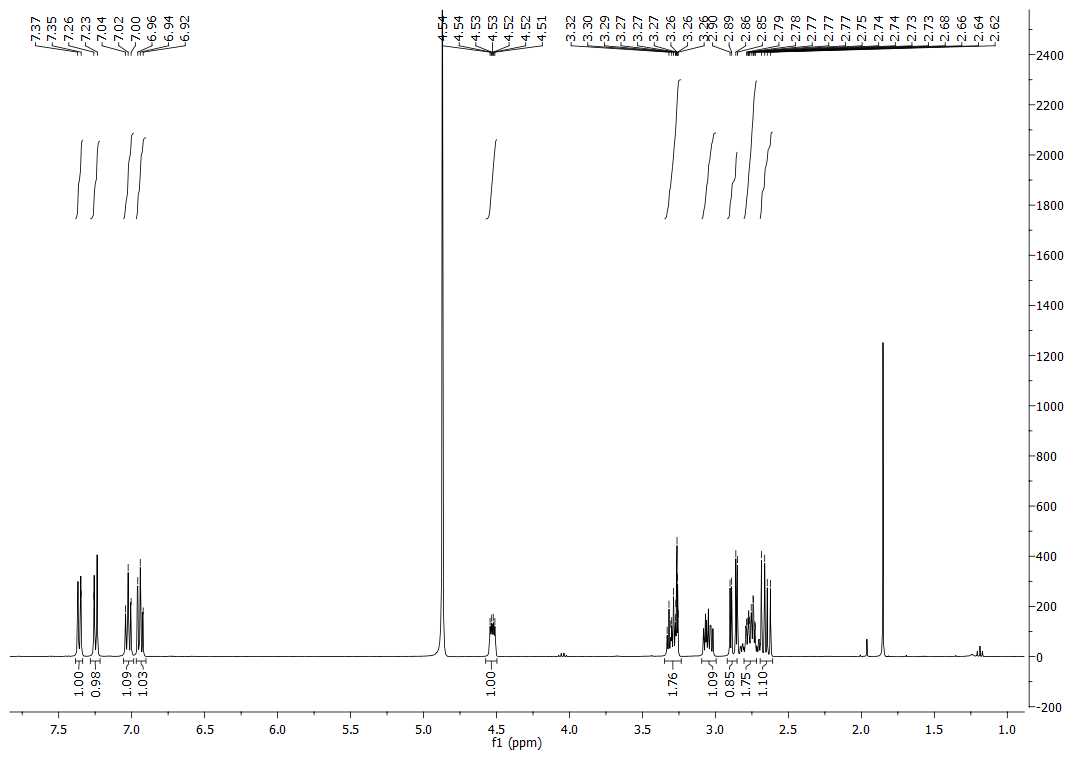


**Supplementary Figure 12**. ^1^H-NMR of compound 1b (MeOD, 400 MHz)

The data are consistent with the published procedure. (K. Diker, K. El Biach, M. Döé de Maindreville e J. Lévy, *J. Nat. Prod.,* **1997**, *60*, 791-793.)

**Chemical synthesis of fragment 1a**

In a flamed-dry 2-necked round-bottom flask, a solution of **1b** (50 mg, 0.22 mmol) in 1 ml of dry THF was added dropwise to a solution of LiAlH_4_ (42.7 mg, 1.12 mmol) in 2.5 ml of dry-THF at 0 °C. The mixture was stirred at reflux for 4 hours and checked by LC-MS; then it was cooled a room temperature and quenched by adding a mixture of THF/H_2_O 4:1. The mixture was then dried over MgSO_4_, filtered on celite-pad, and concentrated at reduced pressure. The crude product was purified twice on RP-HPLC (C18-column, gradient: MeCN + 0.1% FA / H_2_O 3:97 to 20:80 in 15 min) to give a colorless oil with 95% of purity (22% yield).

^1^H NMR (400 MHz, D_2_O) δ 8.48 (br s, 2H), 7.67 (dt, *J* = 7.8, 0.9 Hz, 1H), 7.55 (dt, *J* = 8.1, 0.9 Hz, 1H), 7.37-7.32 (m, 1H), 7.27-7.23 (m, 1H), 4.95 (dd, *J* = 7.8, 5.0 Hz, 1H), 3.82-3.76 (m, 1H), 3.66-3.56 (m, 2H), 3.35-3.31 (m, 2H), 3.18-3.13 (m, 2H), 2.66-2.57 (m, 1H), 2.51-2.41 (m, 1H).


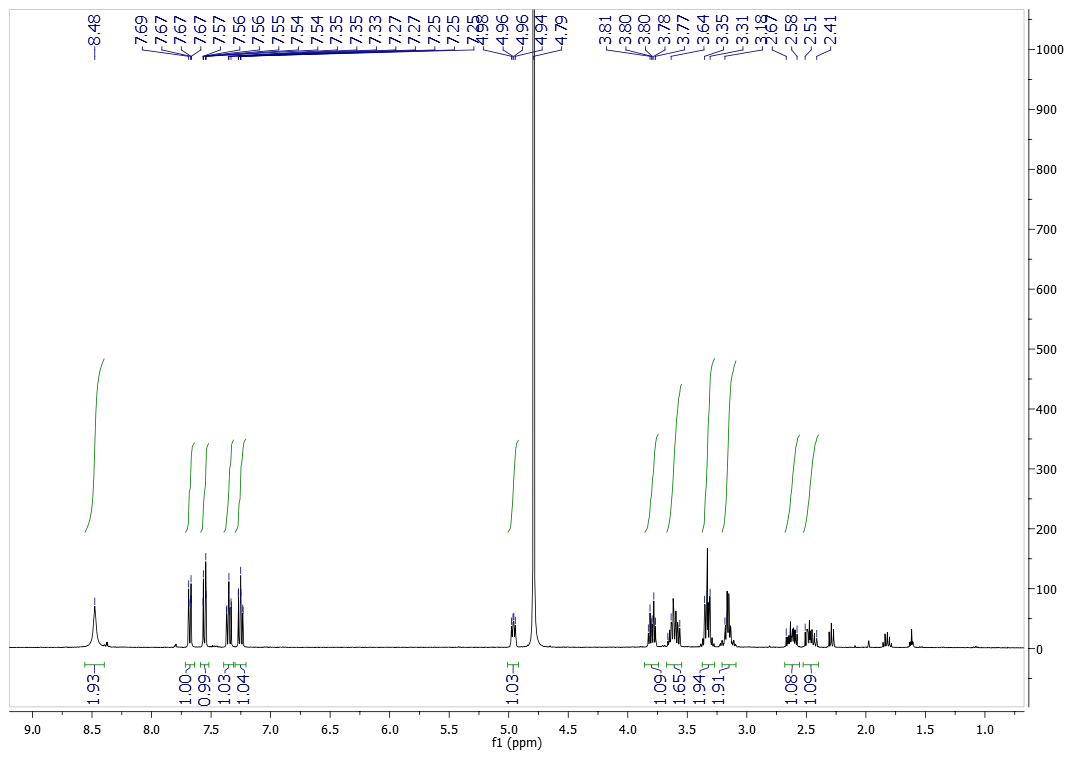


**Supplementary Figure 13.** ^1^H-NMR of compound 1a (D_2_O, 400 MHz)

The data are consistent with the published procedure.

(K. Diker, K. El Biach, M. Döé de Maindreville e J. Lévy, *J. Nat. Prod.,* **1997**, *60*, 791-793.)
